# Growth/differentiation factor 15 causes TGFβ-activated kinase 1-dependent muscle atrophy in pulmonary arterial hypertension

**DOI:** 10.1136/thoraxjnl-2017-211440

**Published:** 2018-12-15

**Authors:** Benjamin E Garfield, Alexi Crosby, Dongmin Shao, Peiran Yang, Cai Read, Steven Sawiak, Stephen Moore, Lisa Parfitt, Carl Harries, Martin Rice, Richard Paul, Mark L Ormiston, Nicholas W Morrell, Michael I Polkey, Stephen John Wort, Paul R Kemp

**Affiliations:** 1 National Heart and Lung Institute, Imperial College London, London, UK; 2 National Pulmonary Hypertension Service, Royal Brompton Hospital, London, UK; 3 Department of Medicine, Addenbrooke’s Hospital, University of Cambridge School of Clinical Medicine, Cambridge, UK; 4 NIHR Respiratory Biomedical Research Unit at the Royal Brompton and Harefield NHS Foundation Trust and Imperial College London, London, UK; 5 Departments of Biomedical and Molecular Sciences, Medicine and Surgery, Queen’s University, Kingston, Ontario, Canada

**Keywords:** primary pulmonary hypertension, exercise

## Abstract

**Introduction:**

Skeletal muscle dysfunction is a clinically important complication of pulmonary arterial hypertension (PAH). Growth/differentiation factor 15 (GDF-15), a prognostic marker in PAH, has been associated with muscle loss in other conditions. We aimed to define the associations of GDF-15 and muscle wasting in PAH, to assess its utility as a biomarker of muscle loss and to investigate its downstream signalling pathway as a therapeutic target.

**Methods:**

GDF-15 levels and measures of muscle size and strength were analysed in the monocrotaline (MCT) rat, Sugen/hypoxia mouse and in 30 patients with PAH. In C2C12 myotubes the downstream targets of GDF-15 were identified. The pathway elucidated was then antagonised in vivo.

**Results:**

Circulating GDF-15 levels correlated with tibialis anterior (TA) muscle fibre diameter in the MCT rat (Pearson r=−0.61, p=0.003). In patients with PAH, plasma GDF-15 levels of <564 pg/L predicted those with preserved muscle strength with a sensitivity and specificity of ≥80%. In vitro GDF-15 stimulated an increase in phosphorylation of TGFβ-activated kinase 1 (TAK1). Antagonising TAK1, with 5(Z)-7-oxozeaenol, in vitro and in vivo led to an increase in fibre diameter and a reduction in mRNA expression of atrogin-1 in both C2C12 cells and in the TA of animals who continued to grow. Circulating GDF-15 levels were also reduced in those animals which responded to treatment.

**Conclusions:**

Circulating GDF-15 is a biomarker of muscle loss in PAH that is responsive to treatment. TAK1 inhibition shows promise as a method by which muscle atrophy may be directly prevented in PAH.

**Trial registration number:**

NCT01847716; Results.

**Key messages**

**What is the key question?**

- What is the association between growth/differentiation factor 15 (GDF-15) and muscle wasting in pulmonary arterial hypertension?

**What is the bottom line?**

- GDF-15 is a responsive biomarker for muscle wasting in pulmonary arterial hypertension that acts through TGFβ-activated kinase 1, a potential target for direct therapeutic intervention.

**Why read on?**

- GDF-15 is a prognostic marker in a wide range of conditions. Here we shed new light on ways in which we might use GDF-15 to identify those at increased risk of muscle wasting and on ways of treating this ubiquitous outcome of chronic disease.

## Introduction

Even with the emergence of modern pulmonary vasodilators the morbidity and mortality for patients with pulmonary arterial hypertension (PAH) remains high.^1^ Skeletal muscle dysfunction is as an important complication and target for therapeutic intervention in patients with PAH.^2 3^ Despite the effects of rehabilitation,^4^ there remains a rationale for developing anabolic agents^5^ which might improve patients’ outcomes.

Muscle strength and size is determined by the balance between protein synthesis and breakdown.^6^ In the quadriceps of patients with PAH there is an upregulation of the proatrophic ubiquitin ligases atrogin-1 and Muscle RING finger protein-1 (MuRF1), while prohypertrophic protein kinase B (AKT),^3^ a component of the insulin-like growth factor 1 (IGF-1) pathway,^6^ is downregulated.^3^ The monocrotaline (MCT) rat, a model of PAH, has smaller hindlimb muscle fibre cross sectional area (CSA), a reduction in contractile function and a tendency to overexpress atrogin-1 and MuRF1, when compared with control treated animals.^7 8^

The transforming growth factor β (TGFβ) proteins have been implicated in the development of both PAH and muscle wasting.^9 10^ One TGFβ protein, growth/differentiation factor (GDF-15) has been shown to be a prognostic marker in idiopathic (I)PAH.^11^ GDF-15 has also been associated with muscle wasting in intensive care unit-acquired weakness (ICUAW), COPD and cancer.^12–15^ GDF-15 has been shown to suppresses appetite,^16^ via its recently discovered receptor the glial cell line-derived neurotrophic factor family receptor-alpha-like (GFRAL)^17^ as well as directly affecting muscle by increasing the local expression of atrogin-1 and MuRF1.^12 13^ The downstream signalling pathway through which GDF-15 acts in muscle remains to be elucidated,^15^ although one potential target is the TGFβ-activated kinase 1 (TAK1), nuclear factor κB (NFκB) pathway.^18^ Delineating and then targeting the pathways through which GDF-15 acts in muscle is a potential method to prevent or treat muscle wasting in PAH as well as other conditions where GDF-15 is a marker of prognosis.

We therefore aimed to determine the local and systemic expression profiles of GDF-15 in animal models of PAH, to investigate the association of GDF-15 and muscle wasting in patients with PAH and to explore the mechanism of action of GDF-15 with a view to developing a therapeutic intervention.

## Methods

The online supplementary material contains full experimental details.

10.1136/thoraxjnl-2017-211440.supp1 Supplementary file 1

### Animal experiments

Animal experiments were approved by the local animal welfare ethics committee and the Home Office (PPL 70/8850).

#### Observational study

Thirty male Sprague-Dawley rats received a single subcutaneous injection of MCT (40 mg/kg) (n=16) or vehicle control (n=14). Food intake was monitored on a per cage basis. Animals were humanely killed at 4 weeks, immediately after MRI (n=3 each group) and haemodynamic assessment.^19^

#### 5(Z)-7-oxozeaenol (Merck Millipore) study

Twenty rats were treated with MCT and 10 with control injections as above. After 2 weeks 10 of the animals in the MCT group were treated with 5(Z)-7-oxozeanol 0.5 mg/kg/day intraperitoneally for 9 days, after which they were humanely killed.^19 20^ One animal in the MCT TAK1i group was excluded from analysis as it had to be humanely killed before the treatment end point.

#### Sugen/hypoxia mouse model

Five Sugen/hypoxia, five hypoxic and five normoxic control mice were treated as previously described.^21^ They were humanly killed after 3 weeks.

### Cell culture and fibre diameter measurement

C2C12 mouse myoblasts were differentiated into myotubes for 10 days. Some myotubes were transfected with pCAGGS-EGFP as previously described.^12^ Mature myotubes were treated for a range of durations with control or GDF-15 (5 or 50 ng/mL) (R&D Systems) with or without 5(Z)-7-oxozeanol (100 nM) (Tocris Bioscience) or MG-132 (10 µM) (Tocris Bioscience) after which cells were lysed for qPCR or western blot or fibre diameter was measured.^12 13 22^

### Tissue processing and qPCR, western blot and ELISA

Muscle and lung samples from rats and mice were homogenised.^13^ qPCR^13^ (primer sequences in the online supplementary table E1) and western blotting^22^ (antibodies in the online supplementary material) were performed according to standard practice. GDF-15 protein levels were determined in rat serum and lung homogenates by ELISA (R&D Systems).

### Fibre diameter measurement and immunohistochemistry

Tibialis anterior (TA) muscle fibre diameter from each animal was analysed.^13 23^ In the observational study, ice crystal damage meant samples were available in 11/16 MCT rats and 12/14 control animals. Sections of lung tissue and TA from five control and five MCT-treated rats were analysed for GDF-15, and in the case of the lung, smooth muscle actin expression.^22^

### Human study

Ethical approval was granted by the REC (13/LO0481) and by the Royal Brompton Hospital (www.clinicaltrials.gov NCT01847716). In 30 patients with PAH, recruited from the Royal Brompton Hospital, we measured quadriceps maximal volitional capacity (QMVC) and rectus femoris cross-sectional area (RF_CSA_) concurrently.^24 25^ QMVC was expressed as a function of body mass index (BMI).^26^ All patients initially enrolled also had their physical activity level (PAL) assessed with the SenseWear Armband. Twenty-five had adequate PAL data.^27^ All patients also underwent 6 min walk distance (6MWD),^28^ echocardiographic assessment and had blood taken for brain natriuretic peptide (BNP) and GDF-15 levels.

### Statistics

Statistics were calculated using GraphPad Prism (V.5, USA). Data are presented as mean±SD or median and IQR. Student’s t-test, Mann-Whitney U test, Pearson or Spearman analysis, Fisher’s exact test and analysis of variance (ANOVA) or repeated measures ANOVA with Bonferroni correction or Kruskal-Wallis with Dunn’s analysis were used to compare groups, dependent on the type and distribution of the data. For correlations strong associations were defined as those with an r>0.5. The proportion of muscle fibres in 5 μm increments was analysed in MCT 5(Z)-7-oxozeaenol experiment as previously described.^23^ Receiver operating characteristic (ROC) curves were constructed for: rats continuing to grow (reaching their maximum weight on the last day of the experiment and not plateaued); patients with retained muscle strength (QMVC/BMI>1.5—based on hospitalisation data from the same cohort, online supplementary figure E1).

## Results

### The MCT rat and Sugen/hypoxia mouse models of pulmonary hypertension are associated with muscle loss

All MCT rats and Sugen/hypoxia mice developed pulmonary hypertension (PH) (online supplementary figure E2). MCT rats weighed, grew and ate less than controls. Although both the MCT rat TA and soleus exhibited muscle loss only the TA and not the soleus atrophy index (muscle weight/total weight) was reduced (online supplementary figure E3). Further investigation was, therefore, limited to the TA. TA size, by MRI and fibre diameter, was lower in the MCT rats compared with controls (online supplementary figure E4). Sugen/hypoxia treated mice also had smaller TAs and TA muscle fibres than hypoxia or control treated animals (online supplementary figure E5).

### Muscle loss in the MCT rat and Sugen/hypoxia mouse is associated with raised circulating GDF-15 levels

**Figure 1:**
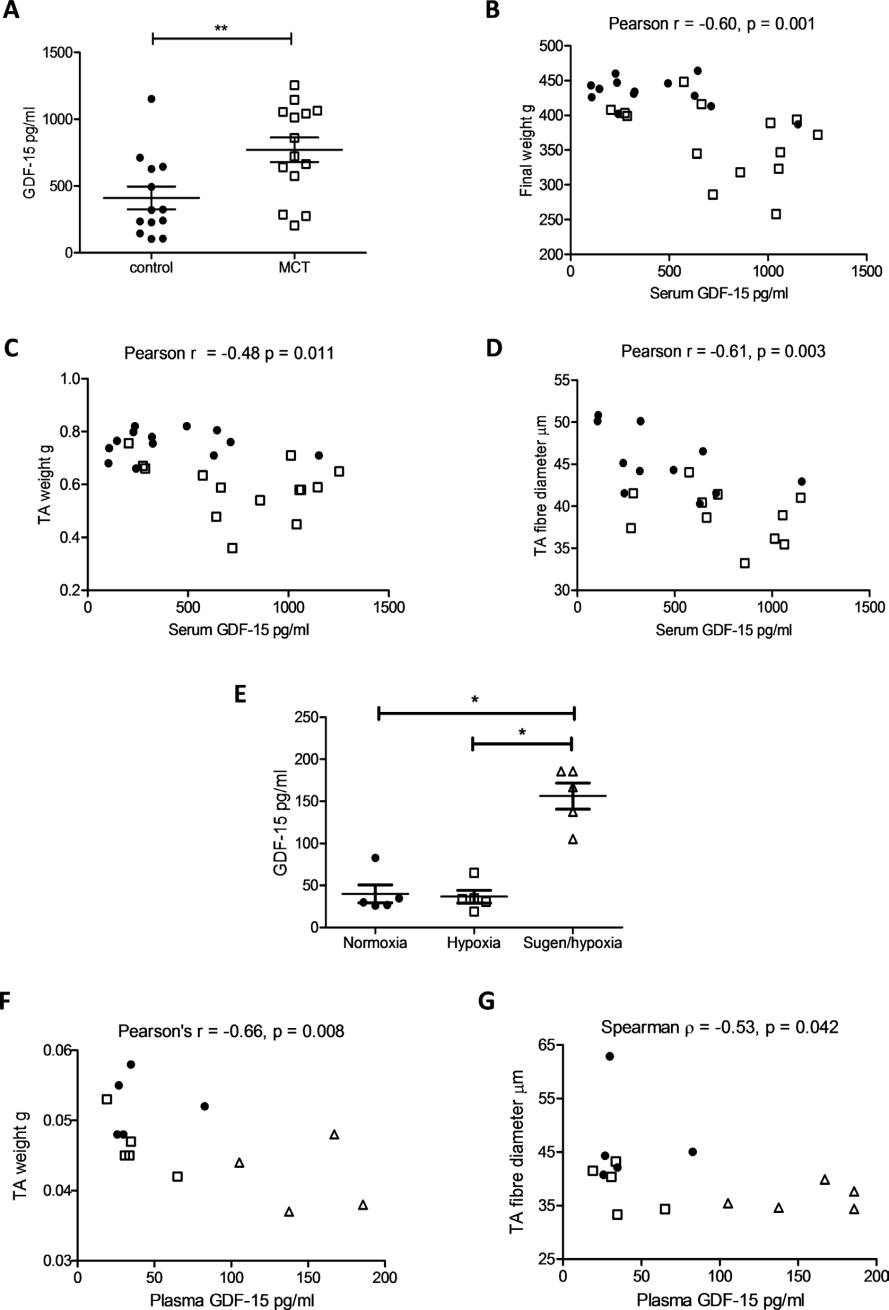
Circulating growth and differentiation factor 15 (GDF-15) is associated with muscle loss in the monocrotaline (MCT) rat and the Sugen/hypoxia mouse. (A) GDF-15 levels in the serum of control (● n=13) and MCT (□ n=14) treated rats (Student’s t-test, p=0.008). (B) Serum GDF-15 levels plotted against final animal weight in control (● n=13) and MCT (□ n=14) treated rats (Pearson r=−0.60 (−0.80 to −0.28), p=0.001). (C) Serum GDF-15 levels plotted against tibialis anterior (TA) weight in control (● n=13) and MCT (□ n=14) treated rats (Pearson r=−0.48 (−0.73 to −0.12), p=0.011). (D) Serum GDF-15 levels plotted against TA fibre diameter in control (● n=11) and MCT (□ n=11) treated rats (Pearson r=−0.61 (−0.82 to −0.25), p=0.003). (E) Plasma GDF-15 levels were higher in the Sugen/hypoxia mouse than in mice held in normoxic or hypoxic conditions (Kruskal-Wallis with Dunn’s correction, p=0.009). (F) Plasma GDF-15 levels plotted against TA weight in grams (g) in control (● n=5), hypoxic (□ n=5) and Sugen/hypoxia (∆ n=5) treated mice (Pearson r=−0.66 (−0.87 to −0.22), p=0.008). (G) Plasma GDF-15 levels plotted against TA fibre diameter in control (● n=5), hypoxic (□ n=5) and Sugen/hypoxia (∆ n=5) treated mice (Spearman r=−0.53 (−0.83 to −0.01), p=0.041).

Serum GDF-15 levels were raised in MCT-treated rats when compared with controls. GDF-15 levels were negatively correlated with final animal weight, TA weight and TA fibre diameter (figure 1A–D). Circulating GDF-15 levels were also raised in the Sugen/hypoxia mice when compared with hypoxia and control treated animals. In this model, both TA weight and fibre diameter were significantly negatively correlated with plasma GDF-15 levels (figure 1E–G). Neither the MCT rat nor the Sugen/hypoxia mouse exhibited any difference in TA GDF-15 expression compared with controls (online supplementary figure E6).

### Circulating GDF-15 in the MCT rat and Sugen/hypoxia mouse correlates closely with lung mRNA expression

**Figure 2:**
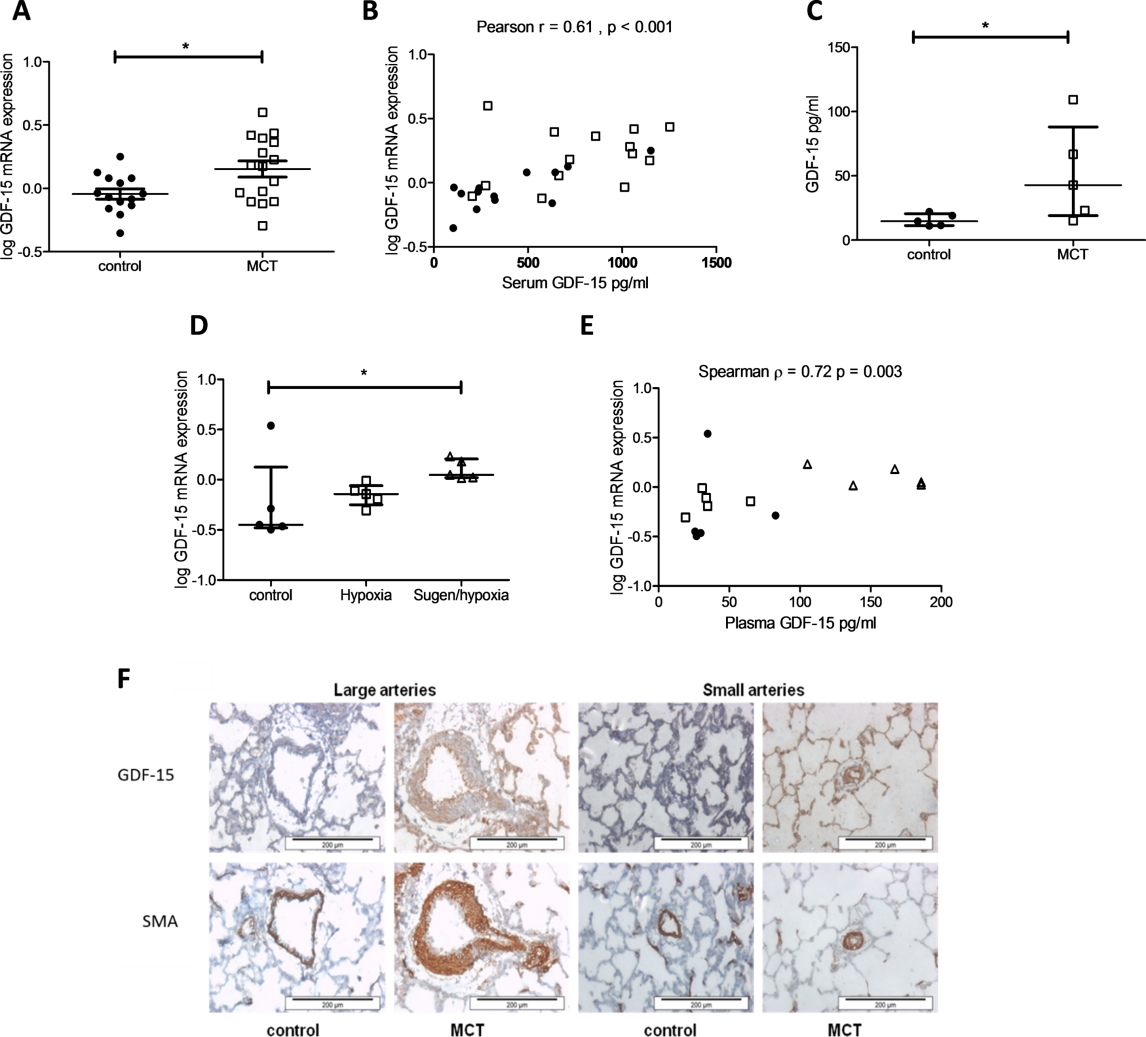
A major source of growth and differentiation factor 15 (GDF-15) in the monocrotaline (MCT) rat and Sugen/hypoxia mouse is likely to be the lung. (A) GDF-15 mRNA expression in the lung of control (n=14) and MCT (n=16) treated rats (Student’s t-test, p=0.017). (B) Serum GDF-15 levels plotted against GDF-15 mRNA expression in the lung of control (● n=13) and MCT (□ n=14) treated rats (Pearson r=0.61 (0.30–0.80), p<0.001). (C) GDF-15 protein expression normalised to loading 100 µg of protein in the lung of control (n=5) and MCT (n=5) treated rats (Mann-Whitney U test, p=0.032). (D) GDF-15 mRNA expression in the lung of control (n=5), hypoxia (n=5) and Sugen/hypoxia (n=5) (Kruskal-Wallis with Dunn’s correction, p=0.042). (E) Serum GDF-15 levels plotted against GDF-15 mRNA expression in the lung of control (● n=5), hypoxia (□ n=5) and Sugen/hypoxia (∆ n=5) mice (Spearman r=0.72 (0.31–0.90), p=0.003). (F) Representative immunohistochemistry of lung sections showing large and small pulmonary arteries stained for GDF-15 or smooth muscle actin (SMA) in control (n=5) and MCT-treated rats (n=5).

GDF-15 mRNA expression was increased in the lung of MCT rats. Lung GDF-15 mRNA expression correlated with serum GDF-15 levels in these animals. GDF-15 protein levels were also raised in the MCT rat lung compared with controls (figure 2A–C). GDF-15 mRNA expression was elevated in the lung of the Sugen/hypoxia mouse, when compared with hypoxia and control treated animals. Here lung GDF-15 mRNA expression also correlated with circulating levels (figure 2D,E). Immunohistochemistry revealed increased staining for GDF-15 in the pulmonary arteries of MCT rats, with staining concentrated in the endothelial cells (figure 2F). In the MCT model GDF-15 levels correlated significantly with RV/LV+S wt but not with right ventricular systolic pressure (RVSP). There was no significant correlation between GDF-15 and these measures in the mouse model (online supplementary figure E7).

### Plasma GDF-15 is associated with muscle strength and size in patients with PAH

**Figure 3:**
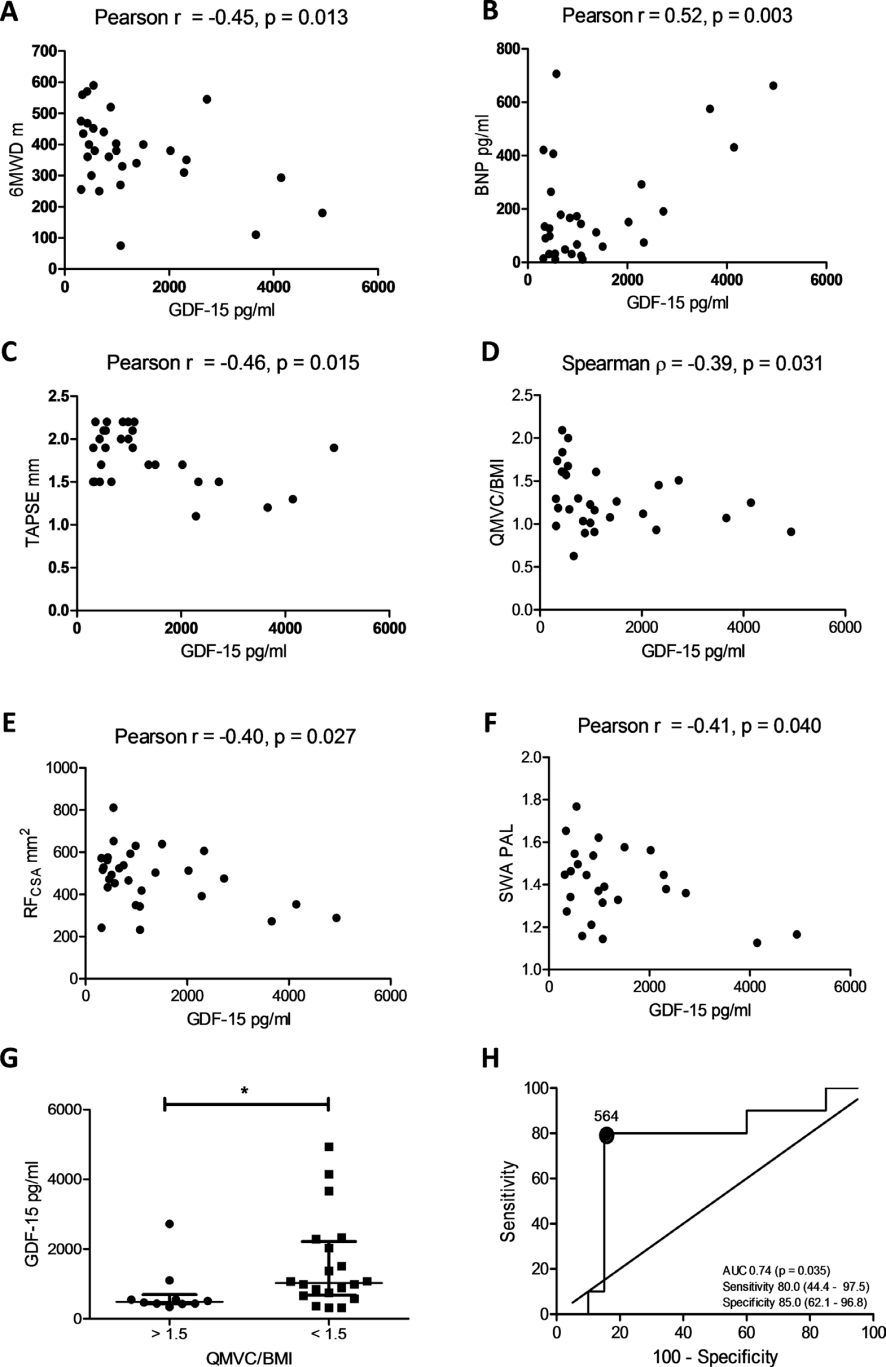
Circulating growth/differentiation factor 15 (GDF-15), muscle wasting and physical activity in patients with pulmonary arterial hypertension (PAH). (A) 6 min walk distance (6MWD) plotted against plasma GDF-15 levels in 30 patients with PAH (Pearson r=−0.45 (−0.69 to −0.10), p=0.013). (B) Brain natriuretic peptide (BNP) plotted against plasma GDF-15 levels in 30 patients with PAH (Pearson r=0.52 (0.19–0.74), p=0.003). (C) Tricuspid annular plane systolic excursion (TAPSE) plotted against plasma GDF-15 levels in 28 patients with PAH (Pearson r=−0.46 (−0.71 to −0.10), p=0.015). (D) Quadriceps maximal volitional capacity (QMVC)/body mass index (BMI) plotted against plasma GDF-15 levels in 30 patients with PAH (Spearman r=−0.39 (−0.67 to −0.03), p=0.031). (E) Ultrasound measured rectus femoris cross-sectional area (USRF_CSA_) plotted against plasma GDF-15 levels in 30 patients with PAH (Pearson r=−0.40 (−0.67 to −0.05), p=0.027). (F) SenseWear Armband (SWA) measured physical activity level (PAL) plotted against plasma GDF-15 levels in 25 patients with PAH (Pearson r=−0.41 (−0.69 to −0.02), p=0.040). (G) GDF-15 levels in patients with high and low muscle strength defined by a QMVC/BMI < or >1.5 (Mann-Whitney U test, p=0.037). (H) Receiver operating characteristic (ROC) curve of GDF-15’s ability to predict those with a QMVC<1.5 or >1.5 (area under the curve (AUC) 0.74 (0.54–0.94), p=0.035).

Patient demographics are described in table 1. Plasma GDF-15 levels correlated with 6MWD, BNP levels and tricuspid anterior posterior systolic excursion (figure 3A–C). No other echocardiographic marker correlated with GDF-15 levels. GDF-15 levels also correlated negatively with QMVC/BMI, RF_CSA_ and PAL (figure 3D–F). GDF-15 levels were significantly lower in those patients with a QMVC/BMI>1.5 when compared with those with a QMVC/BMI<1.5 (figure 3G). GDF-15 levels were able to predict those patients with preserved muscle strength, with an area under the curve (AUC) of 0.74 (0.53–0.94, p=0.035) with levels of <564 pg/L having a sensitivity and specificity of ≥80% (figure 3H).

**Table 1:** Demographic details of 30 patients with pulmonary arterial hypertension investigated for circulating growth/differentiation factor 15 (GDF-15) levels and muscle loss

Demographics	n=30
Age	46.8 (±13.9) years
Female	20
Height	167 (±8) cm
Weight	67.3 (58.8–77.7) kg
Diagnosis	
IPAH	23
Congenital heart disease	7
Echocardiogram	
RVSP	88.5 (±26.8) mm Hg
TAPSE	1.80 (±1.33) mm
TR velocity	408 (±84) cm/s
PAcT	83.8 (±26.5) ms
Exercise, WHO, BNP, GDF-15	
6MWD	373 (±126) m
6MWD % predicted	60.5 (±19.0) %
WHO I: II: III	3: 19: 8
BNP	131 (44, 271) pg/mL
GDF-15	862 (459, 1635) pg/mL

The table presents data as mean and SD or median and IQR dependent on the distribution of the data.

6MWD, 6 min walk distance; BNP, brain natriuretic peptide; GDF-15, growth/differentiation factor 15; IPAH, idiopathic pulmonary arterial hypertension; PAcT, pulmonary artery acceleration time; RVSP, right ventricular systolic pressure (measured by echocardiogram); TAPSE, tricuspid anterior posterior systolic excursion; TR, tricuspid regurgitation; WHO, WHO functional status.

### GDF-15 is proatrophic, activates TAK1 and can be antagonised by TAK1 inhibition

**Figure 4:**
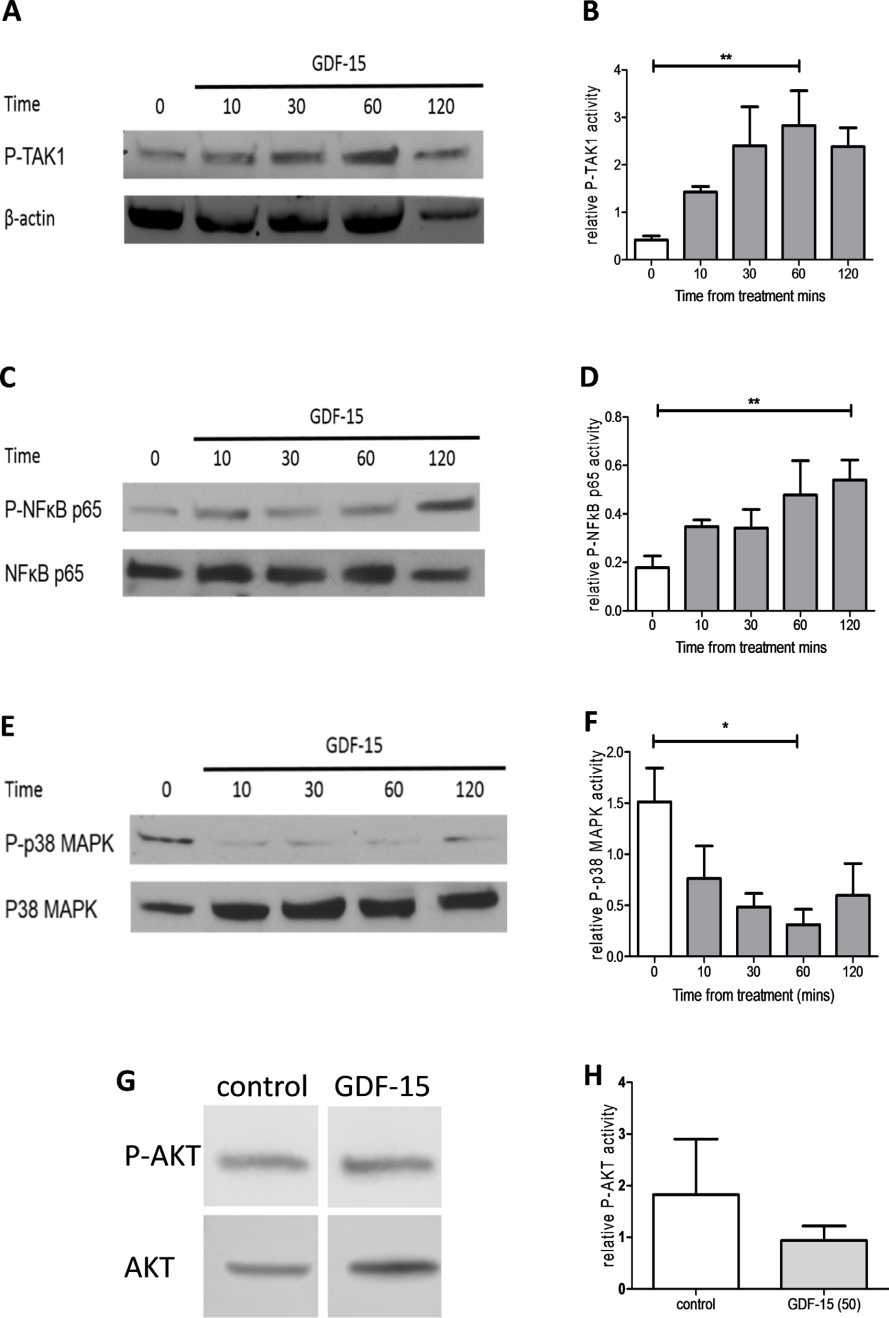
Phosphorylation of transforming growth factor β-activated kinase 1 (TAK1) and its downstream mediators by growth/differentiation factor 15 (GDF-15). Ten-day C2C12 myotubes were treated with GDF-15 (50 ng/mL) for 10, 30, 60 or 120 min. (A) Phosphorylated TAK1 (P-TAK1) and β actin levels (representative blot n=4). (B) Relative intensity of P-TAK1 normalised to β-actin (repeated measures ANOVA, p=0.020). (C) Phosphorylated p65 NFκB (P-NFκB) and total NFκB levels (representative blot n=4). (D) Relative intensity of P-NFκB normalised to total NFκB (repeated measures analysis of variance (ANOVA), p=0.046). (E) Phosphorylated p38 MAPK (P-p38 MAPK) and total p38 MAPK levels (representative blot, n=4). (F) Relative intensity of P-p38 MAPK normalised to total p38 MAPK (repeated measures ANOVA, p<0.001). (G) Phosphorylated protein kinase B (P-AKT) and total AKT levels treated for 60 min with control or GDF-15 50 ng/mL (representative blot, n=7). (H) Relative intensity of P-AKT normalised to total AKT (Student’s t-test, p=0.453).

**Figure 5:**
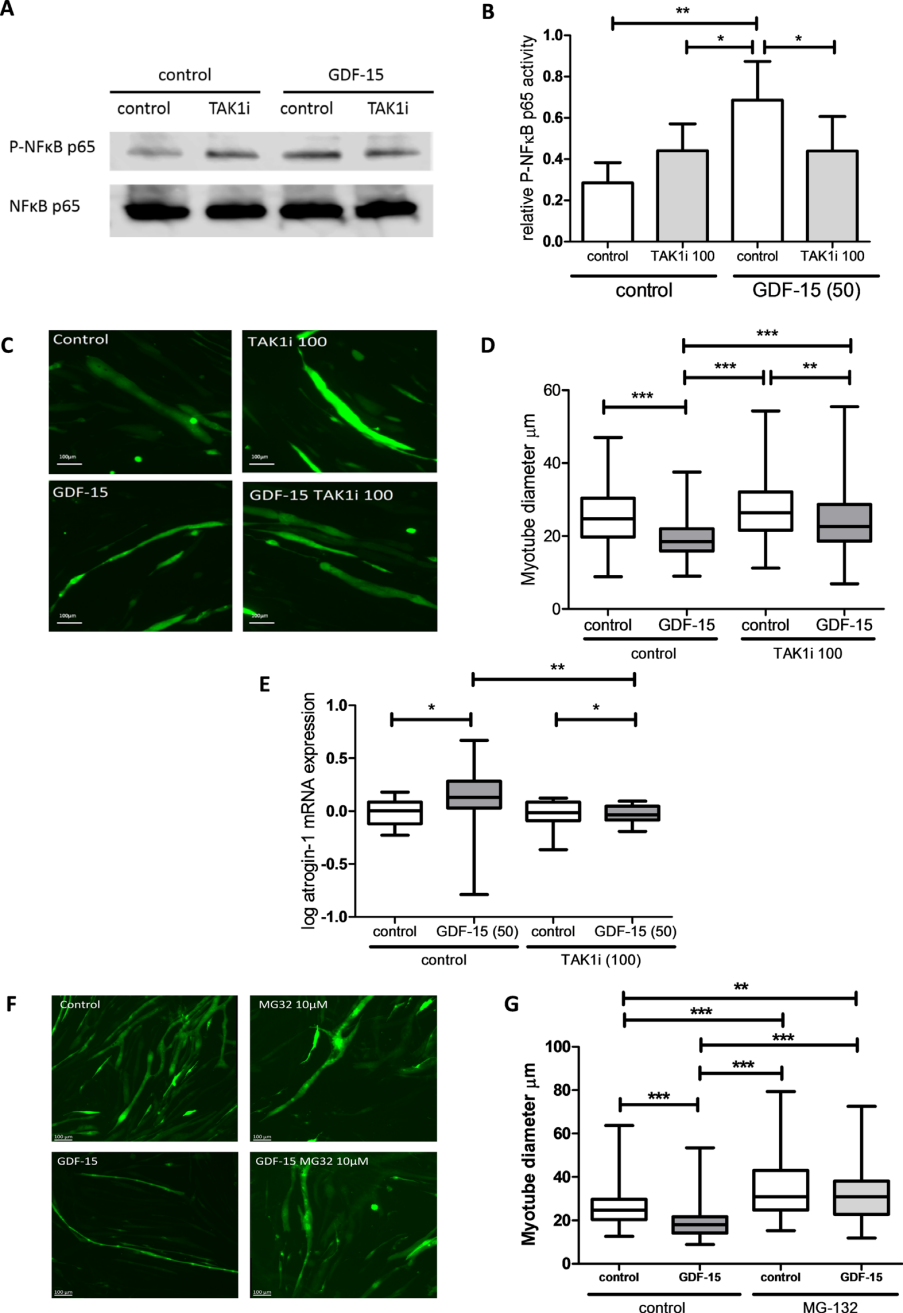
Transforming growth factor β-activated kinase 1 (TAK1) inhibition prevents growth/differentiation factor 15 (GDF-15) mediated atrophy in vitro. (A) Phosphorylated p65 NFκB (P-NFκB) and total NFκB levels in C2C12 myotubes treated with GDF-15 (50 ng/mL) with or without the TAK1 inhibitor 5(Z)-7-oxozeanol (100 nM) for 1 hour (representative blot, n=4). (B) Relative intensity of P-NFκB normalised to total NFκB (repeated measures analysis of variance (ANOVA), p=0.003). (C) Representative green fluorescent protein (GFP) fluorescent images of C2C12 myotubes transfected with pCAGGS-EGFP treated with GDF-15 (50 ng/mL) with or without the TAK1 inhibitor 5(Z)-7-oxozeanol (100 nM) for 48 hours (n=3 experiments). (D) Diameter of C2C12 myotubes transfected with pCAGGS-EGFP treated with GDF-15 (50 ng/mL) with or without the TAK1 inhibitor 5(Z)-7-oxozeanol (100 nM) for 48 hours (Kruskal-Wallis with Dunn’s analysis, p<0.001, n=3 experiments, median number of cells measured per group per experiment, 189 (180–219)). (E) Log atrogin-1 mRNA levels in C2C12 myotubes treated with GDF-15 (50 ng/mL) with or without the TAK1 inhibitor 5(Z)-7-oxozeanol (100 nM) for 96 hours (Kruskal-Wallis with Dunn’s analysis, p=0.026, n=5 experiments in triplicate). (F) Representative GFP fluorescent images of C2C12 myotubes transfected with pCAGGS-EGFP treated with GDF-15 (50 ng/mL) with or without the 26S ribosome inhibitor MG-132 (10 µM) for 48 hours (n=3 experiments). (G) Diameter of C2C12 myotubes transfected with pCAGGS-EGFP treated with GDF-15 (50 ng/mL) with or without the 26S ribosome inhibitor MG-132 (10 µM) for 48 hours (Kruskal-Wallis with Dunn’s analysis, p<0.001, n=3 experiments, median number of cells measured per group per experiment, 141 (88–150)).

In C2C12 myotubes, GDF-15 treatment induced a significant increase in phosphorylation of both TAK1 and its target NFκB, it reduced phosphorylation of p38 mitogen-activated protein kinase and did not affect AKT phosphorylation (figure 4A–H). GDF-15 mediated NFκB phosphorylation was inhibited by the selective TAK1 inhibitor 5(Z)-7-oxozeaenol at a concentration of 100 nM (figure 5A,B). This inhibitor was also able to reverse the atrophic effects of GDF-15 on myotube diameter (figure 5C-D) as well as prevent increases in mRNA expression of atrogin-1 (figure 5E). The addition of MG-132, a 26S proteasome inhibitor, to myotubes also prevented GDF-15 mediated reductions in myotube diameter, suggesting that GDF-15 has direct proteolytic effects on muscle (figure 5F,G). The effects of GDF-15 at 5 ng/mL on phosphorylation of TAK1 and NFκB and myotube diameter were similar to those seen with 50 ng/mL (online supplementary figure E8).

### Downstream targets of GDF-15 are overexpressed in the TA of the MCT rat

**Figure 6:**
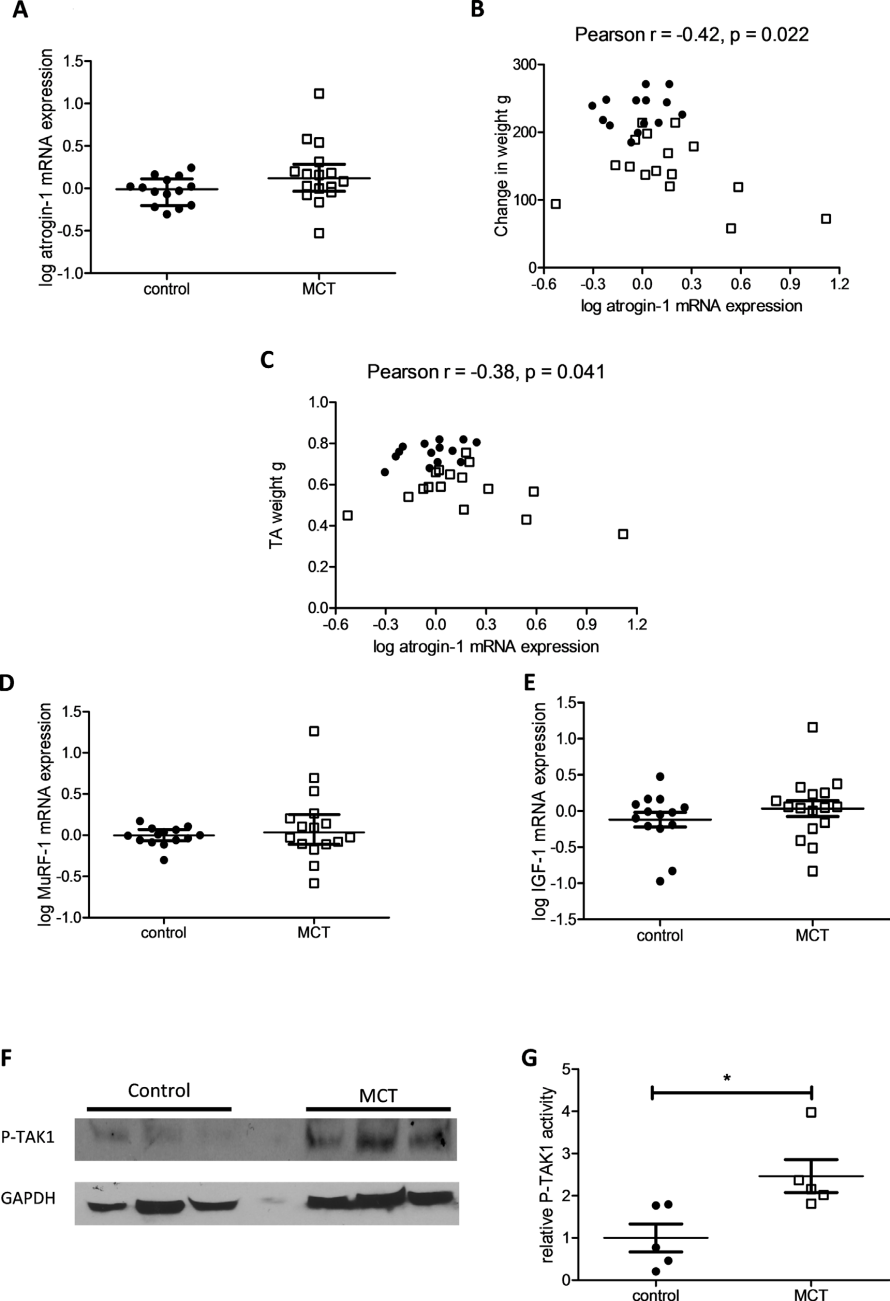
Downstream targets of growth/differentiation factor 15 (GDF-15) are overexpressed in the tibialis anterior (TA) of the monocrotaline (MCT) rat. (A) Log atrogin-1 mRNA expression in the TA of the MCT (n=16) and control (n=14) treated rats (Mann-Whitney U test, p=0.077). (B) TA log atrogin-1 mRNA expression plotted against change in weight in MCT (□ n=16) and control (● n=14) treated rats (Pearson r=−0.42 (−0.68 to −0.07), p=0.022). (C) TA log atrogin-1 mRNA expression plotted against TA weight in MCT (□ n=16) and control (● n=14) treated rats (Pearson r=−0.38 (−0.65 to −0.02), p=0.041). (D) Log MuRF-1 mRNA expression in the TA of the MCT (□ n=16) and control (● n=14) treated rats (Mann-Whitney U test, p=0.329). (E) Log IGF-1 mRNA expression in the TA of the MCT (□ n=16) and control (□ n=14) treated rats (Student’s t-test, p=0.317). (F) Western blot of phospho-TAK1 (P-TAK1) and GAPDH in the TA of the MCT (□ n=5) and control (● n=5) treated rats (representative blot). (G) P-TAK1 protein levels normalised to GAPDH levels in the TA of the MCT and control treated rats (n=5, Student’s t-test, p=0.021).

There was a trend to an increased mRNA expression of atrogin-1 in the TA of the MCT rat compared with control (p=0.077) (figure 6A). TA atrogin-1 mRNA levels correlated negatively and significantly with animal growth and TA weight (figure 6B,C). There was no difference in MuRF-1 or prohypertrophic IGF-1 mRNA expression in the TA of the MCT rats (figure 6D,E). MCT/control rats, in the interventional study, also had significantly increased levels of phospho-TAK1 within their TA when compared with control/control treated animals (figure 6F,G).

### TAK1 inhibition prevented muscle loss in some MCT-treated rats

**Figure 7:**
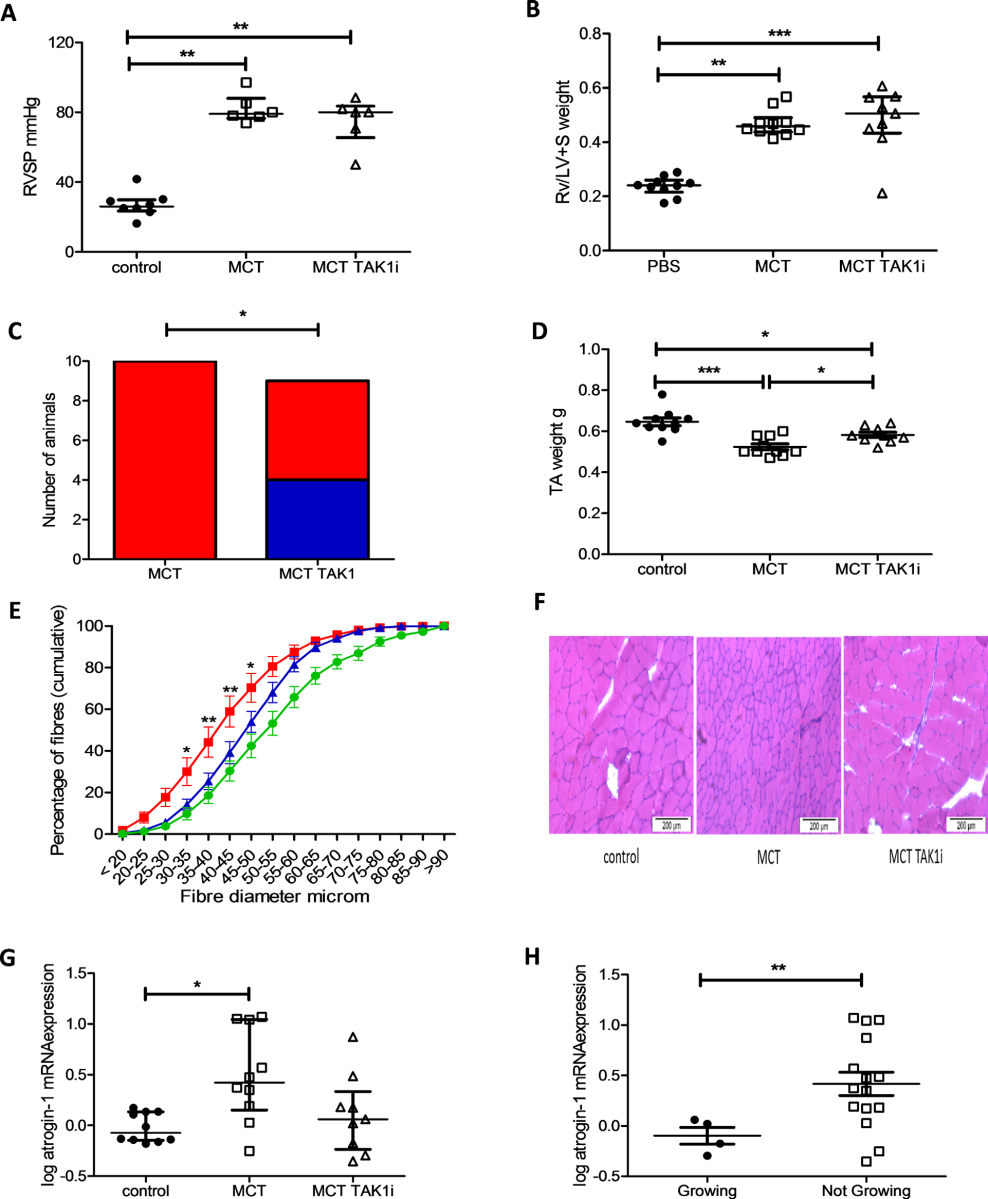
Transforming growth factor β-activated kinase 1 (TAK1) inhibition prevents tibialis anterior (TA) muscle atrophy in the monocrotaline (MCT) rat as well as preventing weight loss in some animals. (A) Right ventricular systolic pressure (RVSP) in control (● n=8), MCT (□ n=6) and MCT TAK1i (∆ n=6) treated rats (Kruskal-Wallis with Dunn’s correction, p=0.001). (B) Right ventricle/left ventricle plus septal (RV/LV+S) weight in control (● n=10), MCT (□ n=10) and MCT TAK1i (∆ n=9) treated rats (Kruskal-Wallis with Dunn’s correction, p<0.001). (C) Number of MCT animals growing and not growing at the end of the experiment in the MCT (n=10) and MCT TAK1i (n=9) groups (Fisher’s exact test, p=0.033). (D) TA weight in control (● n=10), MCT (□ n=10) and MCT TAK1i (∆ n=9) treated rats (one-way analysis of variance (ANOVA) with Bonferroni correction, p<0.001). (E) Fibre profiles of control (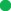
n=10), MCT (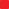
n=10) and MCT TAK1i (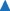
n=9) plotted as mean and SEM of proportion of fibres below the indicated fibre diameter in 5 µm increments (two-way ANOVA, p<0.001 for row, column and interaction, * and ** represent a significant difference between MCT and MCT TAK1 proportions at each 5 µm fibre intervals by Bonferroni). (F) Representative bright-field image of rat TA muscle tissue stained with H&E from which median fibre diameter was determined. (G) Log atrogin-1 mRNA expression in the TA of control (● n=10), MCT (□ n=10) and MCT TAK1i (∆ n=9) treated rats (Kruskal-Wallis with Dunn’s correction, p=0.018). (H) Log atrogin-1 mRNA expression in the TA of MCT-treated rats growing (● n=4) and not growing (□ n=15) at the end of the experiment (Mann-Whitney U test, p=0.032).

TAK1 inhibition had no effect on RVSP or RV/LV+S wt (figure 7A,B). In this experiment, 10/10 MCT/control and 4/9 MCT/TAK1i-treated rats had stopped growing by the end of the experiment (figure 7C). MCT/TAK1i treated rats had higher TA weights as well as a higher proportion of larger TA fibres than MCT/control treated animals (figure 7D–F). MCT/TAK1i rats tended to have lower TA atrogin-1 mRNA expression than MCT/control animals, a change that did not meet statistical significance (figure 7G). There was no change in MuRF-1 mRNA expression in these animals (online supplementary figure E9). MCT rats that continued to grow exhibited lower atrogin-1 mRNA expression in their TA than MCT rats that were not growing (figure 7H). In this experiment, there was also no difference in average food intake per animal between the MCT/control and MCT/TAK1i-treated animals (388 g vs 386 g, respectively).

### Circulating GDF-15 is a biomarker of muscle loss that is responsive to treatment

**Figure 8:**
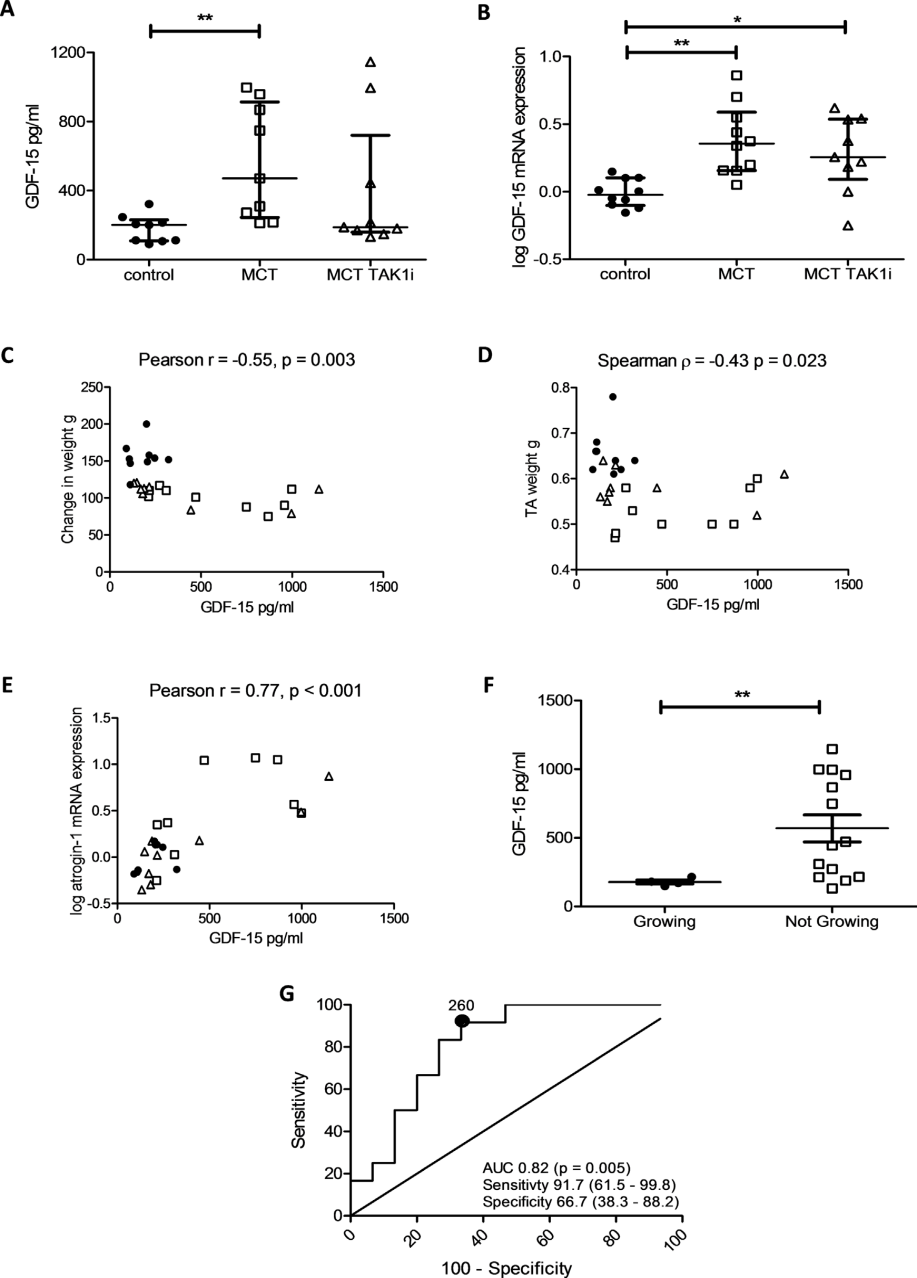
Growth/differentiation factor 15 (GDF-15) is a marker of muscle loss in the monocrotaline (MCT) rat that responds to treatment. (A) Circulating GDF-15 in pg/mL in control (● n=9), MCT (□ n=9) and MCT TAK1i (∆ n=9) treated rats (Kruskal-Wallis, p=0.013). (B) Log GDF-15 mRNA expression in the lung of control (● n=10), MCT (□ n=10) and MCT TAK1i (∆ n=9) rats (Kruskal-Wallis, p=0.002). (C) Plasma GDF-15 levels plotted against changed in weight in grams (g) in control (● n=9), MCT (□ n=9) and MCT TAK1i (∆ n=9) treated rats (Pearson r=−0.55 (−0.77 to −0.22), p=0.003). (D) Plasma GDF-15 levels plotted against tibialis anterior (TA) weight in grams (g) in control (● n=9), MCT (□ n=9) and MCT TAK1i (∆ n=9) treated rats (Spearman r=−0.43 (−0.71 to −0.06), p=0.023). (E) Plasma GDF-15 levels plotted against log atrogin-1 mRNA expression in control (● n=9), MCT (□ n=9) and MCT TAK1i (∆ n=9) treated rats (Pearson r=−0.77 (0.55–0.89), p<0.001). (F) GDF-15 levels in MCT-treated rats growing (● n=4) and not growing (□ n=14) at the end of the experiment (Mann-Whitney U test, p=0.022). (G) Receiver operating characteristic (ROC) curve of GDF-15’s ability to predict those still growing at the end of the experiment across all animals (n=27) (area under the curve (AUC) 0.82 (0.65–0.98), p=0.005).

There was a trend to a reduction in circulating GDF-15 levels in MCT/TAK1i-treated animals that did not meet statistical significance (471 (244, 913) in MCT/control vs 188 (159, 720) in MCT/TAK1i). This trend to reduction in GDF-15 levels was mirrored in the mRNA from lung homogenates (0.356 (0.156, 0.588) in MCT/control vs 0.256 (0.092, 0.537) in MCT/TAK1i) (figure 8A,B). Across this experiment circulating GDF-15 levels correlated negatively with change in animal weight and TA weight and positively with TA atrogin-1 mRNA expression (figure 8C–E). In those MCT-treated animals still growing at the end of the experiment there was a significant reduction in circulating GDF-15 level (figure 8F). An ROC curve for GDF-15 in predicting those animals growing and not growing at the end of the experiment had an AUC of 0.82 (0.65–0.97, p=0.005) (figure 8G). The AUC when assessing GDF-15’s utility in determining those animals growing and not growing in the MCT rats alone was 0.89 (0.74–1.0, p=0.020) (online supplementary figure E10).

## Discussion

We have shown that GDF-15 levels may be a useful biomarker that is responsive to treatment aimed at preventing muscle loss in PAH. Our data also implicate proteolytic pathways including TAK1-NFκB and atrogin-1 in GDF-15 meditated muscle wasting, a process that can be at least partially reversed by the TAK1 inhibitor 5(Z)-7-oxozeanol.

The role of TGFβ proteins in muscle wasting in PAH has not been widely analysed, being limited to one study showing increased circulating myostatin in MCT-treated rats.^29^ We have identified, as is the case in ICUAW,^12^ COPD^14^ and cancer,^30^ that PAH-associated muscle loss may also be a GDF-15 driven phenomenon.

Studies in ICUAW have supported a potential autocrine or paracrine effect of GDF-15 on muscle mass.^13^ A GDF-15 antibody has been shown to be able to prevent the muscle loss seen in mice xenografted with human tumours which secreted GDF-15 confirming its endocrine effects.^30^ Our data also suggest that GDF-15 mediates muscle loss in an endocrine manner, with the pulmonary vascular endothelium and plexiform lesions^31^ acting as the potential source of raised circulating GDF-15 levels.

We have previously shown that GDF-15 can cause an increase in the expression of the ubiquitin ligases atrogin-1 and MuRF-1 in myotubes in culture.^13^ Our study adds weight to the argument that GDF-15 causes muscle loss through proteolysis and not through inhibition of the prohypertrophic IGF-1-AKT pathway. It also seems that the TAK1 pathway may play a pivotal role in the muscle wasting process stimulated by GDF-15 and its inhibition rescues GDF-15 mediated atrophy in cells through a reduction in atrogin-1 mRNA.

GDF-15 has previously been shown to signal through TAK1 and NFκB in *Escherichia coli* infected epithelial cells,^18^ but not previously in muscle cells. TAK1 is a key intermediate in a number of signalling pathways that drive muscle wasting including myostatin and tumour necrosis factor α.^9 32^ Clinical trials of drugs targeted at the muscle aiming to block these factors individually have met with limited success.^33 34^ 5(Z)-7-oxozeanol has been used to antagonise TAK1 in a number of animal models of disease including autoimmune diabetes^35^ and cerebral ischaemia.^20^ TAK1 inhibitors may be more successful than other more specific drugs aimed at antagonising muscle loss, as they have the potential to inhibit these synergistic muscle wasting pathways.^29^

Recent data suggest that TAK1 knockout in satellite stem cells prevents regeneration after acute muscle injury resulting in a significant reduction in muscle mass both in vivo and in vitro.^36^ Our data suggest that rather than inhibiting growth TAK1 inhibition results in an increase in muscle size in the MCT rat. This may be due to the small doses of 5(Z)-7-oxozeaonal used, which might result in a reduction but not abolition of TAK1 signalling. It is also likely that it reflects differences in the activity of TAK1 on satellite cells and mature myofibres and the predominant processes leading to loss of muscle in the various models. For example, inhibiting satellite cell proliferation/differentiation is likely to have a large effect on muscle mass in a model of regeneration in response to injury but less effect on a model of atrophy.

An ideal biomarker should be specific, easily detectable, obtained relatively non-invasively, related to disease severity and disease progression, including response to treatment, and be measurable in a standardised way.^37^ We have shown that in MCT-related muscle loss, GDF-15 is able to fulfil all these criteria. GDF-15 has been identified as a biomarker and potential regulator of muscle wasting in a number of conditions^12 14^ and PAH is no different. It is now important to test whether patients who gain muscle mass in PAH have a GDF-15 profile similar to the MCT rats who responded most significantly to 5(Z)-7-oxozeaenol treatment.

Johnen *et al* reported that GDF-15 can reduce muscle mass by suppressing appetite.^16^ Consistent with this, MCT-treated animals in our study ate significantly less than their control counterparts. However, TAK1 inhibition had no discernible effect on food intake in the MCT rat while it did affect muscle mass. This, along with in vitro data, suggests that TAK1, a downstream mediator of GDF-15, has a direct proatrophic effect on skeletal muscle that is independent of its role as an appetite suppressant. This is supported by data in ICUAW, where, circulating GDF-15 levels were associated with muscle loss, despite that patients being fed by nasogastric tube^12^ and the fact that the GFRAL receptor through which GDF-15 controls appetite^17^ is not present in the skeletal muscle.^38^ Overall, it seems most likely that GDF-15 affects muscle mass both directly and also through appetite suppression; however, in both cases inhibiting GDF-15 activity is likely to reduce muscle wasting.

Our data have potential implications outside PAH. GDF-15 has been repeatedly identified as a marker of mortality in a wide range of health and disease.^39^ Sarcopenia and cachexia are also almost universally associated with increased risk of morbidity and mortality across disease states.^40^ Our data add more evidence suggesting that GDF-15 and its downstream signalling molecule TAK1 may be a potential target for future therapeutic intervention aimed at improving muscle mass, exercise tolerance, quality of life and possibly even mortality across a wide range of conditions.

### Limitations

There was wide variation in the induction of PH in the animal studies, which when combined with the relatively low number of samples studied and the requirement to analyse specific time points, may have contributed to the absence of statistical significance for some of the observations. Alternative sources of GDF-15 production outside the lung and muscle contributing to the systemic levels of the protein have not been excluded. This is especially true of experiments involving the systemic toxin MCT. The majority of the western blots were performed on film, where the relationship between band density and protein content is not linear. The clinical study is cross sectional, was conducted in a single, heterogeneous population, the ROC curve is unvalidated and the animal model studies are associative, which can only suggest, but not prove, a causal link between GDF-15 and muscle loss in PAH. Finally, the relative contributions of appetite, high levels of GDF-15 and low physical activity to muscle loss in PAH have not been defined and remain speculative. In particular, in those with very low functional capacity, inactivity is likely to be a marked contributor to muscle loss. Future work showing that a reduction in GDF-15 levels is associated with improved muscle mass as well as functional outcomes would add weight to our argument.

## Conclusions

GDF-15 has been shown to be a candidate ‘ideal’ biomarker of muscle and weight loss in PAH. The lung tissue was shown to be a potential source of circulating GDF-15. GDF-15’s effects on muscle in vitro appear to be mediated by proteolysis and the activation of TAK1 since inhibition of both the 26S ribosomal protein unit and the kinase, by a specific inhibitor, suppressed GDF-15-induced myotube atrophy, making TAK1 inhibition an attractive target to prevent muscle loss in PAH. This study also has wider implications for the investigation and management of muscle loss across the spectrum of human disease, where GDF-15 may be important.
