# Supplementary material for: Growth/differentiation factor 15 causes TGFβ-activated kinase 1-dependent muscle atrophy in pulmonary arterial hypertension
Source: Thorax. 2018 Dec 15;74(2):164–76. doi: 10.1136/thoraxjnl-2017-211440 (PMC6467240; doi:10.1136/thoraxjnl-2017-211440)
Supplement: Supplementary file 1 [file thoraxjnl-2017-211440supp001.pdf]

Supplementary Material

Methods

Animal experiments

All animal experiments were undertaken at University of Cambridge and approved by both the local animal welfare ethics committee and the Home Office under license PPL 70/8850.

Male Sprague-Dawley rats (Charles River Laboratories, Harlow, UK), (6 to 7 weeks old) received a single subcutaneous injection of MCT (Sigma-Aldrich, St-Louis, MO) (40 mg/kg) or vehicle control. Food intake was monitored throughout the experiment on a per cage basis with 2-4 similarly treated rats per cage. To characterise the muscle loss associated with PAH animals were humanely killed at 4 weeks, immediately after haemodynamic assessment.

In the TAK1 inhibitor (TAK1i) study 20 rats were treated with MCT (40mg/kg) and 10 with control injections as above. After 2 weeks 10 of the animals in the MCT group were treated with 5(Z)-7-oxozeanol (Merck Millipore, Watford, UK) 0.5mg/kg/day (dissolved in 10% DMSO/90% PBS) intra-peritoneally for 9 days as previously described [1]. Control and the other 10 MCT animals were treated with 10% DMSO/90% PBS for the same length of time. One animal in the MCT TAK1i group before starting had to be humanely killed 2 days before completing the full course of 5(Z)-7-oxozeanol. This animal was therefore excluded from further analysis, leaving 9 rats in the MCT TAK1i group.

Male C57BL/6J mice (Charles River Laboratories, Harlow, UK), (11 weeks old) were injected subcutaneously with SU5416 (Sugen) (Sigma-Aldrich, St-Louis, MO) (20 mg/kg) or vehicle control on a weekly basis. Mice were exposed to either room air or chronic normobaric hypoxia inside a ventilated plexiglass chamber as previously described [2]. To characterise the muscle loss associated with PAH animals were humanely killed after

3 weeks, immediately after haemodynamic assessment. Normoxic mice did not undergo haemodynamic assessment so RVSP and RV/LV+S weight in these animals was compared to standard normal values derived from Tabima et al. [3].

Rats and mice were exsanguinated and blood was collected in SST or EDTA coated tubes and later spun down for removal of serum or plasma which was stored at -80^{0}C. Half of the lungs were rendered in a distended state by infusion of low melting point agarose into the trachea for 1 minute and then were placed in 4% paraformaldehyde before being embedded in paraffin. The remaining lungs were immediately frozen in liquid nitrogen for protein and RNA isolation. The TA and soleus muscles were identified and dissected out bilaterally and weighed. One was immediately frozen in liquid nitrogen the other was cut longitudinally, placed in OCT on a cork and then frozen in isopentane dipped in liquid nitrogen (MCT) or placed in formaldehyde (Sugen/hypoxia) for future analysis.

Hemodynamic Evaluation and right ventricular hypertrophy (RVH)

Rats and mice were anesthetized with isoflurane for hemodynamic assessment at the end of the experiments. Body weight was recorded, and right-heart catheterization was performed using a 1.4F microtip transducing catheter (Millar Instruments, Houston, TX) as previously described [3, 4]. To assess the extent of RVH the heart was removed, and the RV free wall was dissected from the LV+S and weighed separately; the degree of RVH was determined from the ratio RV/LV+S.

MRI (full details)

Three rats in each group were anaesthetised with isoflurane (2-3% in 1l/min O2). MRI was performed at 4.7T using a Bruker BioSpec 47/40 system with an 86mm transmit/receive coil provided by the manufacturer (Bruker Inc., Germany). To separate signals from water and

fat contributions, a three-point Dixon method based on IDEAL [5] was used with echo times of 5.44ms, 5.70ms and 6.21ms and a repetition of time of 12ms. The flip angle was 20° and the receiver bandwidth was 50kHz. Images were reconstructed using Matlab (Mathworks, Cambridge, UK) using iterative estimation of local field deviations to produce images showing water and fat.

C2C12 cell culture and transfection

C2C12 mouse myoblasts were differentiated into myotubes for 10 days. Prior to differentiation myoblasts for fibre diameter experiments were transfected with pCAGGS-EGFP using lipofectamine (Life Technologies, Carlsbad, CA, USA) as previously described [6]. Mature myotubes were treated for 48 hours (for fibre diameter) or 96 hours (for qPCR) with control or GDF-15 (50ng/ml) (R&D systems, Abingdon, UK) with or without 5(Z)-7-oxozeanol (100nM) (Tocris bioscience, Avonmouth, Bristol, UK) or MG-132 (10 µM) (Tocris bioscience, Avonmouth, Bristol, UK) after which myotube diameter was measured or cells were harvested for qPCR using trizol (Life Technologies, Carlsbad, CA, USA), chlorophorm, isopropanol extraction protocols as previously described [6, 7]. For phospho-protein detection cultured myotubes were serum starved overnight and then treated GDF-15 (50ng/ml) (R&D systems, Abingdon, UK) at a number of different time points, after which they were lysed with cell lysis buffer (CLB) and PMSF (Cell signalling technology, Danvers, MA, USA). Protein content was analysed using Bio-rad protein assay reagent (Biorad, Herts, UK) read on a plate reader (Biotek, Swindon, UK) at 595nm and compared to known protein contents of bovine serum albumin standards (Sigma Aldrich, St Louis, MA, USA). Protein was then stored in loading buffer and DTT (Cell signalling technology, Danvers, MA, USA) for western blot.

Tissue processing for qPCR and ELISA

Muscle and lung samples were homogenised using Precellys 24™ tissue homogeniser (Stretton Scientific, Derbyshire UK) with 1.4mm ceramic beads (CDK-14) in 2ml tubes. Protein was extracted using cell lysis buffer (CLB) and PMSF (Cell signalling technologies, Danvers, MA, USA), whilst RNA was extracted using trizol (Life Technologies, Carlsbad, CA, USA) as described above. Protein content was analysed using a Bradford assay (Biorad, Herts, UK) as described above. Protein for ELISA was stored at -20°C.

qPCR

cDNA was made from mRNA extracted from muscle and lung homogenates and *in vitro* cell culture samples using Omniscript (Qiagen, Hilden, Germany). Complementary Deoxyribonucleic acid (DNA) levels were quantified using Sybr green qPCR (Quantifast® - Qiagen, Hilden, Germany) and an AB 7500 (Fast) qPCR thermal cycler (Life technologies, Carlsbad, CA, USA) as previously described [7]. Cycle threshold (CT) values and melt curves were generated for each PCR product. Each sample was run in duplicate. CT values for mRNA of interest were normalised to the geomean of housekeeper genes RPLPO and GAPDH for cell work and RPLPO for animal experiments. Results were expressed and analysed based on these normalised samples. All primers were bought from Sigma-Aldrich (Sigma Aldrich, St Louis, MA, USA), the sequences of which are listed in Table 1.

Western blotting and ELISA

Samples from cell culture for western blotting were heated to 95°C for 5 minutes and then loaded at various concentrations in 10% acrylamide SDS-PAGE gels. Once the protein had run into the gel it was wet transferred to a membrane (Biorad, Herts, UK).

This was blocked with 5% BSA or 5% milk in 0.05% tris-buffered saline with tween (TBS-T). Membranes were cut at 55KDa so that the membranes could be interrogated for 2 different proteins on the same blot. Membranes were then incubated with primary antibody in BSA or milk overnight at 4°C. The antibodies used were anti-phospho TAK1 (9339), anti-phospho NFκB p65 (3033), anti-phosoho p38 MAPK (9211), anti-phospho AKT (9271) , anti-NFκB p65 (sc-372, Santa Cruz, California, USA), anti- p38MAPK (9212), anti- AKT (9272), anti-β-actin (4967) and anti-GAPDH (2118) (all from Cell signalling technologies, Danvers, MA, USA, unless otherwise stated). Blots were then washed with 0.05% TBS-T and incubated with secondary antibody conjugated with horseradish peroxidase (Cell signalling technologies, Danvers, MA, USA) for 1 hour at room temperature. They were washed again and exposed to Pierce enhanced chemiluminecence (ECL) detection reagent (Thermo-Fischer, Waltham, MA, USA) and imaged on an Ettan dige-imager (EDI) (GE healthcare) or with radiographic film using a photo-developer. Blots were then scanned in using a cannon scanner (Cannon, Surrey, UK) and analysed by densitometry using Image J software. For cell culture samples phospho-TAK1 was normalised to β-actin, whilst phospho NFκB p65 was normalised to total NFκB p65 and phospho p38 MAPK was normalised to total p38 MAPK levels. For rat TA samples both phospho-TAK1 and phospho-NFκB p65 were normalised to total GAPDH. Plasma from patients and mice and serum and lung homogenates from rats had their GDF-15 levels measured by ELISA as per manufacturer's instructions (human GDF-15 ELISA R&D systems / mouse rat GDF-15 ELISA R&D systems, Abingdon, UK).

Immunoflourescence for C2C12 myotube diameter

C2C12 myotubes which had been transfected with pCAGGS-EGFP and treated with GDF-15 with or without 5(Z)-7-oxozeanol as described above for 48 hours were imaged using

a fluorescence microscope using the protocol from Bloch *et al.* [6] with myotubes being measured in 20 randomly selected fields per well. In total between 154 and 227 myotubes were analysed per treatment group across 3 experiments.

Immunohistochemistry and fibre diameter measurement

Tibialis anterior muscle from each animal was cut into 10 micron sections and mounted on slides. The muscle was stained with haematoxylin and eosin. Images were photographed at 10x magnification and fibre diameter measured using Image-J software. The minimal Feret's diameter was measured as described previously [7]. In the MCT rat study adequate samples were available in 11/16 MCT rats and 12/14 vehicle control treated rats, due to ice crystal damage. Data was available from all 10 animals in the Sugen/hypoxia and control mouse group and all 30 animals in the MCT TAK1 inhibitor experiment.

Immunohistochemistry for lung and tibialis anterior GDF-15 expression

Sections of lung tissue embedded in wax from 5 controls and 5 MCT rats were cut into 6 micron sections. These were rehydrated with histoclear (National Diagnostics, Georgia, USA) and ethanol. Sections were boiled with sodium citrate (Sigma-Aldrich, St Louis, MA, USA) for antigen retrieval after which they were incubated in 0.3% H_2O_2 for 20 minutes and blocked in goat serum before being incubated overnight with rabbit anti-GDF-15 (orb49016, 1:100, biorbyt, Cambridge, UK) or anti-smooth muscle actin (M0851, 1:200, DAKO, Ely, UK) at 4°C. Following washing with PBS the sections were incubated with biotinylated anti-rabbit or anti-mouse IgG (BA 1000 / BA 9200, 1:200, Vector labs, Burlingham, CA, USA), respectively, for 30 minutes. Sections were washed and then incubated with avidin and biotinylated enzyme mix (Vectastain, Elite ABC reagent PK-6100 series, Vector labs, Burlingham CA, USA), underwent further washing and were then stained with diaminobenzidine (Sigma-Aldrich, St Louis, MA, USA) and haematoxylin (VWR, Radnor, PA, USA). Slides were mounted and

imaged at 20x magnification. Sections of TA tissue embedded and frozen in OCT from 5 controls and 5 MCT rats were cut into 10 micron sections were incubated in 0.3% H_2O_2 for 20 minutes and blocked in goat serum before being incubated overnight with rabbit anti-GDF-15 (orb49016, 1:100, biorbyt, Cambridge, UK) at 4°C. Following washing with PBS the sections were incubated with biotinylated anti-rabbit (BA 1000, 1:200, Vector labs, Burlingham, CA, USA), for 30 minutes. Sections were washed and then incubated with avidin and biotinylated enzyme mix (Vectastain, Elite ABC reagent PK-6100 series, Vector labs, Burlingham CA, USA), underwent further washing and were then stained with diaminobenzidine (Sigma-Aldrich, St Louis, MA, USA) after which some were counterstained with haematoxylin (VWR, Radnor, PA, USA). Slides were mounted and imaged at 40x magnification.

Patient recruitment and observations.

Ethical approval for the study was granted by the REC (13/LO0481) and by the local research and development department at the Royal Brompton Hospital. The study was registered on www.clinicaltrials.gov (NCT01847716).

PAH patients, who were 16 years and older and in World Health Organisation (WHO) class I - III were eligible for recruitment. Exclusion criteria were: those with significant cardio-respiratory disease; metabolic abnormalities including diabetes, eating disorders or untreated thyroid disease; pre-existing, known causes of muscle weakness or wasting, including, but not limited to, debilitating stroke and neuromuscular disease; active malignancy; and those not able to exercise.

Thirty eligible patients with PAH were recruited from the PH clinic at the Royal Brompton Hospital between 2013 and 2017. (Thirty-six patients were approached; 1 patient refused to

participate; 2 patients were excluded; 3 patients did not have muscle function measured). We measured their quadriceps strength and size concurrently. They had their quadriceps maximal volitional capacity (QMVC) [8] measured which was normalised to their BMI. QMVC is the gold standard for assessment of quadriceps strength. The standard method for assessing QMVC was first described by Edwards et al. It was initially developed for use in patients with COPD and healthy controls. The patient adopted a sitting position on a specially designed chair which was calibrated daily with a 29kg weight. A strap attached to a strain gauge was looped around the participants' dominant leg. The participant was asked to make an isometric voluntary contraction of the quadriceps muscles. The procedure was repeated until the maximum contraction was determined. This was normally the 3rd or 4th attempt. There were no adverse events in the patients with PAH performing this task [8]. Measurements were made using Labchart version 7 (ADI instruments, Oxford, UK). In order to allow for comparison across different individuals with differing heights and weights QMVC was normalised to body mass index. This method has been used in studies by Swallow *et al.* [9] and Canavan *et al.* [10]. A normal QMVC/BMI in healthy non-smokers aged 65 years was 1.45 [11]. Patients also had their rectus femoris cross sectional area(US RFcsa) measured using the ultrasound technique described by Seymour *et al*. [12]. An 8MHz 5.cm linear transducer array in B-mode (PLM805, Toshiba Medical Systems, Crawley, UK) was used to conduct this assessment. The participant lay flat with the dominant leg held in passive extension. A Point 3/5 of the distance between the anterior superior iliac spine and superior boarder of the patella was marked and the measurement of the rectus femoris muscle was made at this point. The technique included the use of contact gel, minimal pressure and anterior posterior motion to help identify the image of the muscle with the smallest cross sectional area. The femur was identified in each image to aid orientation. The

septa that defined the edges of the muscle were identified by voluntary muscle contraction. Three frozen images were taken and the inner echogenic line of the rectus femoris defined by hyperechoic septae was measured using a planimetric technique (Nemio, Toshiba Medical Systems). The average of the 3 $USRF_{CSA}$ measurements that were within 10% of each other was taken and documented. The technique is that described by Seymour *et al* [12] and used in a number of other studies [13-15]. In one study CT of the quadriceps correlated with US RF_{CSA} with a r^2 of 0.55 ($p<0.001$) [12]. A normal US RF_{CSA} in healthy volunteers with an average age of 63 was just under $500mm^2$[12]. The patients underwent 6MWT according to American Thoracic Society standards [16]. 6MWD percent predicted was calculated using formulae by Enright and Sherrill [17]. Patients also underwent echocardiographic assessment of pulmonary pressures and had blood tests taken for routine bloods, BNP as well as GDF-15 levels which were measured by ELISA (R&D systems, Abingdon, UK). All patients had their physical activity measured by the Sensewear armband (SWA) (SenseWear® Pro armband; BodyMedia Inc., Pittsburgh, PA, USA). This is a physical acitivity monitor that is worn over the triceps. It is a biaxial accelerometer, with other sensors including those for galvanic skin response, heat flux, skin temperature, and near-body ambient temperature. It has been validated in healthy individuals [18] and in some patients with chronic disease such as COPD [19]. It has also been used to evaluate activity in patents with PAH [20]. Participants wore the monitor for seven days at all times except when bathing or swimming to avoid the armband getting wet. Physical activity level was the output chosen for analysis as it gives an overall assessment of global physical activity. The physical activity level (PAL: total energy expenditure per day divided by basal metabolic rate) was calculated using minute to minute data generated by the SWA Pro software version 6.1.0.1523 (BodyMedia Inc., Pittsburgh, PA, USA) [21]. In analysis of the data a PAL:

< 1.4 defines extremely inactive individuals, 1.4 -1.69 defines sedentary individuals, 1.70-1.99 defines moderately active individuals, 2.0-2.4 defines vigorously active individuals and >2.4 defines extremely active individuals [22, 23]. Data was excluded from analysis if the SWA was worn for less than an average of 22.5 hours per day for 5 days including at least one weekend day as described by Watz et al [24]. Adequate data was available from 25/30 patients. Twenty-eight patients from this cohort were followed up for up for at least 2 years after their initial assessment, by case note review. Admission was defined as an overnight stay in hospital and was collected over the follow up period.

Statistics

In animal and human experiments differences between controls and PAH groups or those with high and low muscle strength were assessed using student's t-test or Mann-Whitney U test depending on the distribution of the data. Normality was assessed visually using histograms and the Kolmogorov Smirnov test. Correlations were assessed using Pearson or Spearman analysis depending on the distribution and linearity of the association of the data sets. Multiple comparisons were using the same data set not corrected for using a Bonferroni corrections for multiple analysis. This is because the numbers needed to study would be higher than feasibly possible given the time and resources available and the rarity of the condition. This does leave us open to type 1 error but reduces the amount of type 2 error. As long as this is understood by the reader then this seems to be a reasonable approach and is supported in the wider literature when dealing with small sample sizes [25]. Missing data was a problem especially with regard to the SWA. All available data was included in the assessment to maximise the validity of our results, a technique advocated by Dziura *et al.* [26]. In order to pick up an r of -0.5, with an α of 0.05 and a β of 0.2, for correlations between GDF-15 and muscle parameters, the primary outcome for the study, 29 participants were required. Therefore after enrolling 30

participants analysis was undertaken, despite the fact that initially it was felt that double this number were going to be required to show a difference between those with preserved and lost muscle function. ROC curve analysis was used to define the ability of GDF-15 to predict patients with preserved muscle strength defined as a QMVC/BMI of >1.5. This cut-off was chosen based on data showing that a QMVC/BMI of > 1.5 was highly specific in defining those not admitted to hospital within 2 years of enrolment in the study (Figure E1). Rats who continued to grow in the interventional experiment were defined as those who reached their maximum weight on the last day of treatment without plateauing. Fisher's exact test was used to compare the proportion of rats still growing in the MCT and MCT TAK1i groups. In the MCT TAK1 experiments fibre diameter was assessed as an average across each group and as a curve showing proportions of cells below cumulative 5 micron cut-offs, which were then assessed using ANOVA [27]. Strong correlations were considered those with an r value of > 0.5. In graphs as convention * represents a significance level of < 0.05 but > 0.01, ** represents a significance level of < 0.01 but > 0.001, and *** represents a significance of < 0.001.

References

1. White, B.J., et al., *Protection from cerebral ischemia by inhibition of TGFbeta-activated kinase.* Exp Neurol, 2012. **237**(1): p. 238-45.
2. Ciuclan, L., et al., *A novel murine model of severe pulmonary arterial hypertension.* Am J Respir Crit Care Med, 2011. **184**(10): p. 1171-82.
3. Tabima, D.M., T.A. Hacker, and N.C. Chesler, *Measuring right ventricular function in the normal and hypertensive mouse hearts using admittance-derived pressure-volume loops.* Am J Physiol Heart Circ Physiol, 2010. **299**(6): p. H2069-75.
4. Phillips, P.G., et al., *cAMP phosphodiesterase inhibitors potentiate effects of prostacyclin analogs in hypoxic pulmonary vascular remodeling.* Am J Physiol Lung Cell Mol Physiol, 2005. **288**(1): p. L103-15.
5. Reeder, S.B., et al., *Iterative decomposition of water and fat with echo asymmetry and least-squares estimation (IDEAL): application with fast spin-echo imaging.* Magn Reson Med, 2005. **54**(3): p. 636-44.
6. Bloch, S.A., et al., *Sustained elevation of circulating growth and differentiation factor-15 and a dynamic imbalance in mediators of muscle homeostasis are associated with the development of acute muscle wasting following cardiac surgery.* Crit Care Med, 2013. **41**(4): p. 982-9.
7. Bloch, S.A., et al., *Increased expression of GDF-15 may mediate ICU-acquired weakness by down-regulating muscle microRNAs.* Thorax, 2015. **70**(3): p. 219-28.
8. Edwards, R.H., et al., *Human skeletal muscle function: description of tests and normal values.* Clin Sci Mol Med, 1977. **52**(3): p. 283-90.
9. Swallow, E.B., et al., *Quadriceps strength predicts mortality in patients with moderate to severe chronic obstructive pulmonary disease.* Thorax, 2007. **62**(2): p. 115-20.
10. Canavan, J.L., et al., *Functionally Relevant Cut Point for Isometric Quadriceps Muscle Strength in Chronic Respiratory Disease.* Am J Respir Crit Care Med, 2015. **192**(3): p. 395-7.
11. Patel, M.S., et al., *Klotho and smoking--An interplay influencing the skeletal muscle function deficits that occur in COPD.* Respir Med, 2016. **113**: p. 50-6.
12. Seymour, J.M., et al., *Ultrasound measurement of rectus femoris cross-sectional area and the relationship with quadriceps strength in COPD.* Thorax, 2009. **64**(5): p. 418-23.
13. Puthucheary, Z.A., et al., *Acute skeletal muscle wasting in critical illness.* JAMA, 2013. **310**(15): p. 1591-600.
14. Shrikrishna, D., et al., *Quadriceps wasting and physical inactivity in patients with COPD.* Eur Respir J, 2012. **40**(5): p. 1115-22.
15. Maddocks, M., et al., *Neuromuscular electrical stimulation to improve exercise capacity in patients with severe COPD - Authors' reply.* Lancet Respir Med, 2016. **4**(4): p. e16.
16. Laboratories, A.T.S.C.o.P.S.f.C.P.F., *ATS statement: guidelines for the six-minute walk test.* Am J Respir Crit Care Med, 2002. **166**(1): p. 111-7.
17. Enright, P.L. and D.L. Sherrill, *Reference equations for the six-minute walk in healthy adults.* Am J Respir Crit Care Med, 1998. **158**(5 Pt 1): p. 1384-7.
18. Fruin, M.L. and J.W. Rankin, *Validity of a multi-sensor armband in estimating rest and exercise energy expenditure.* Med Sci Sports Exerc, 2004. **36**(6): p. 1063-9.
19. Van Remoortel, H., et al., *Validity of six activity monitors in chronic obstructive pulmonary disease: a comparison with indirect calorimetry.* PLoS One, 2012. **7**(6): p. e39198.
20. Mainguy, V., et al., *Assessment of daily life physical activities in pulmonary arterial hypertension.* PLoS One, 2011. **6**(11): p. e27993.
21. Watz, H., et al., *Extrapulmonary effects of chronic obstructive pulmonary disease on physical activity: a cross-sectional study.* Am J Respir Crit Care Med, 2008. **177**(7): p. 743-51.
22. Manini, T.M., et al., *Daily activity energy expenditure and mortality among older adults.* JAMA, 2006. **296**(2): p. 171-9.

23. Garfield, B.E., et al., *Stanford Seven-Day Physical Activity Recall questionnaire in COPD.* Eur Respir J, 2012. **40**(2): p. 356-62.
24. Watz, H., et al., *Physical activity in patients with COPD.* Eur Respir J, 2009. **33**(2): p. 262-72.
25. Nakagawa, S., *A farewell to Bonferroni: the problems of low statistical power and publication bias.* Behavioral Ecology, 2004. **15**(6): p. 1044-1045.
26. Dziura, J.D., et al., *Strategies for dealing with missing data in clinical trials: from design to analysis.* Yale J Biol Med, 2013. **86**(3): p. 343-58.
27. Lee, J.Y., et al., *FHL1 activates myostatin signalling in skeletal muscle and promotes atrophy.* FEBS Open Bio, 2015. **5**: p. 753-62.

Supplementary Tables and Figures

	Primers
Rat RPLPO F	ATGGGCAAGAACACCATGATG
Rat RPLPO R	CCTCCTTGGTGAACACAAAGC
Rat GDF-15 F	AGCTGTCCGGATACTCAGTC
Rat GDF-15 R	GAGTCTCTTGGGTGCAAATG
Rat atrogin 1 F	CCATCAGGAGAAGTGGATCTATGTT
Rat atrogin 1 R	GCTTCCCCCAAAGTGCAGTA
Rat MuRF-1 F	TGGAGATGAATTGCTCAGT
Rat MuRF-1 R	GTGAAGTTGCCCCCTTACAA
Rat IGF-1 F	TTCATACGCTGTGTGTGGTC
Rat IGF-1 R	CACACTTGGGCACATTTTCT
Mouse RPLPO F	GGACCCGAGAAGACCTCCTT
Mouse RPLPO R	TGCTGCCGTTGTCAAACACC
Mouse GAPDH F	ACTCCACTCCACGGCAAATTCA
Mouse GAPDH R	CGCTCCTGGAAGATGGTGAT
Mouse atrogin-1 F	TCAGCCTCTGCATGATGTTC
Mouse atrogin-1 R	TGGGTGTATCGGATGGAGAC
Mouse MuRF-1 F	CGGGCAACGACCGAGTGCAGACGATC
Mouse MuRF-1 R	CCAGGATGGCGTAGAGGGTGTCAAAC
Mouse GDF-15 F	GGCTGCATGCCAACCAGAG
Mouse GDF-15 R	TCTCACCTCTGGACTGAGTATCC

Table E1. Primers for use in real time qPCR

mRNA transcripts investigated included growth differentiation factor 15 (GDF-15), atrogin-1 muscle ring finger 1 (MuRF-1) and insulin like growth factor 1 (IGF-1) in rat and mouse tissue and C2C12 mouse muscle cells. Housekeeping genes used as normalisers included RPLPO for tissue samples and RPLPO and GAPDH for cell work. All annealing temperatures were set at 60°C.

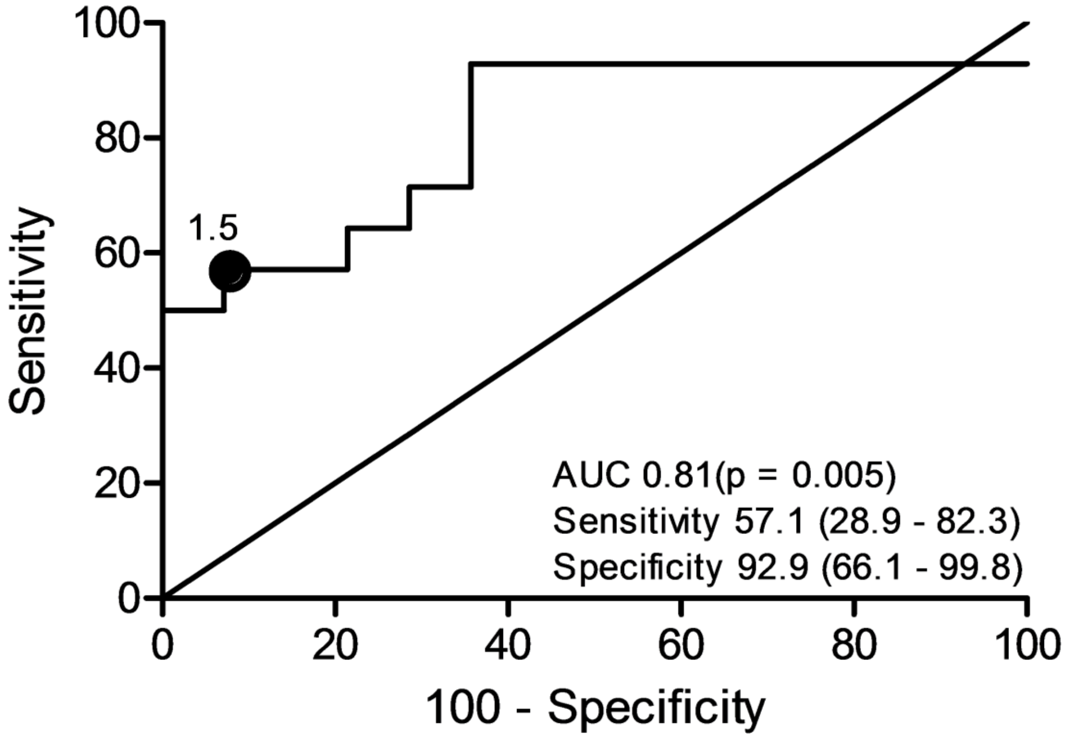

100
80
60
40
20
0
Sensitivity
1.5
AUC 0.81(p = 0.005)
Sensitivity 57.1 (28.9 - 82.3)
Specificity 92.9 (66.1 - 99.8)
0
20
40
60
80
100
100 - Specificity

Figure E1. Quadriceps maximal volitional capacity / body mass index as a predictor of hospital admissions in patients with pulmonary arterial hypertension.

ROC curve of QMVC/BMI in predicting hospital admissions at 2 years (n=28 with follow up data). A QMVC/BMI of > 1.5 was 93% specific in predicting those not admitted to hospital over that time (AUC 0.81 (0.64 – 0.98), p = 0.005).

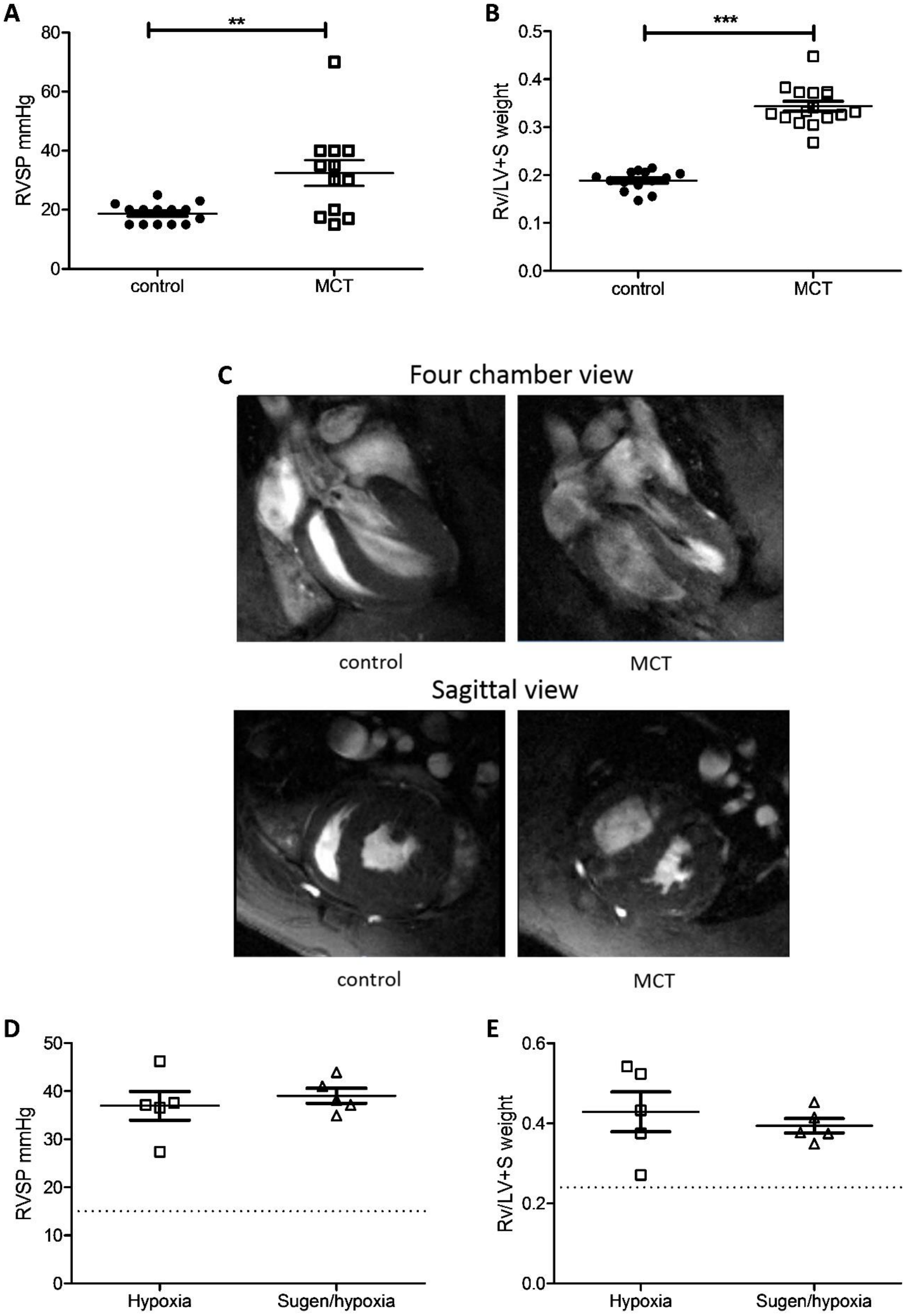
A
**
RVSP mmHg
80
60
40
20
0
control
MCT
B
***
Rv/LV+S weight
0.5
0.4
0.3
0.2
0.1
0.0
control
MCT
C
Four chamber view
control
MCT
Sagittal view
control
MCT
D
RVSP mmHg
50
40
30
20
10
0
Hypoxia
Sugen/hypoxia
E
Rv/LV+S weight
0.6
0.4
0.2
0.0
Hypoxia
Sugen/hypoxia

Figure E2. Pumonary hypertension in the monocrotaline (MCT) rat and the Sugen/hypoxia mouse.

A. Right ventricular systolic pressure (RVSP) (mmHg) in control (● n = 14) and MCT (□ n = 12) treated rats (Student's t-test p = 0.003). **B.** Right ventricle / Left ventricle plus septal weight ration (RV/LV+S) in control (● n = 14) and MCT (□ n = 16) treated rats (Student's t-test $p < 0.001$). **C.** Example magentic resonance images of control (n = 3) and MCT (n = 3) treated rat hearts in systole showing evidence of a hypertrophied and pressure loaded right ventricle in both saggital and 4 chamber view. **D.** Right ventricular systolic pressure (RVSP) (mmHg) in hypoxia (□ n = 5) and Sugen/ hypoxia (Δ n = 5) treated mice (Dotted line represents normal RVSP derived from Tabima et al (2010) [3]). **E.** Right ventricle / Left ventricle plus septal weight ratio (RV/LV+S) in hypoxia (□ n = 5) and Sugen/ hypoxia (Δ n = 5) treated mice (Dotted line represents normal RV/LV+S derived from Tabima et al (2010) [3]).

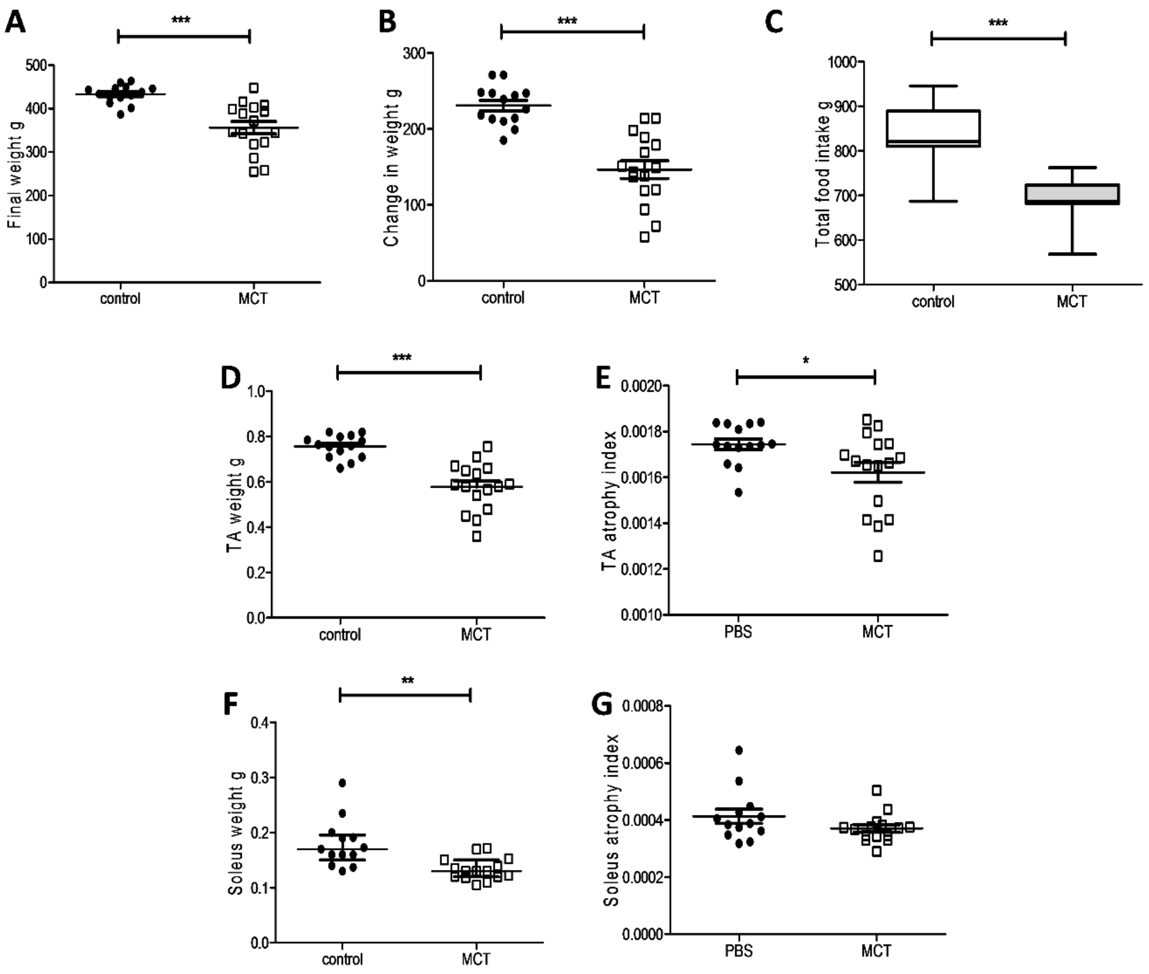
A
***
Final weight g
500
400
300
200
100
0
control
MCT
B
***
Change in weight g
300
200
100
0
control
MCT
C
***
Total food intake g
1000
900
800
700
600
500
control
MCT
D
***
TA weight g
1.0
0.8
0.6
0.4
0.2
0.0
control
MCT
E
*
TA atrophy index
0.0020
0.0018
0.0016
0.0014
0.0012
0.0010
PBS
MCT
F
**
Soleus weight g
0.4
0.3
0.2
0.1
0.0
control
MCT
G
Soleus atrophy index
0.0008
0.0006
0.0004
0.0002
0.0000
PBS
MCT

Figure E3. The monocrotaline (MCT) rat is a model of cardiac cachexia

A. Final weight of control (● n=14) or MCT (□ n=16) treated rats (Student's t-test $p<0.001$). **B.** Change in weight of control (● n=14) or MCT (□ n=16) treated rats (student's t-test $p<0.001$). **C.** Average cage food intake per animal in control or over the 4 weeks after treatment with control or MCT (Mann Whitney U test $p < 0.001$). **D.** Tibialis anterior (TA) muscle weight in control (● n=14) or MCT (□ n=16) treated rats (student's t-test $p < 0.001$). **E.** TA muscle atrophy index (TA weight / total weight) in control (● n=14) or MCT (□ n=16) treated rats (student's t-test $p = 0.022$). **F.** Soleus muscle weight in control (n=14) or MCT (n=16) treated rats (Mann Whitney U test, $p < 0.001$). **G.** Soleus muscle atrophy index (Soleus weight / total weight) in control (● n=14) or MCT (□ n=16) treated rats (student's t-test $p = 0.151$).

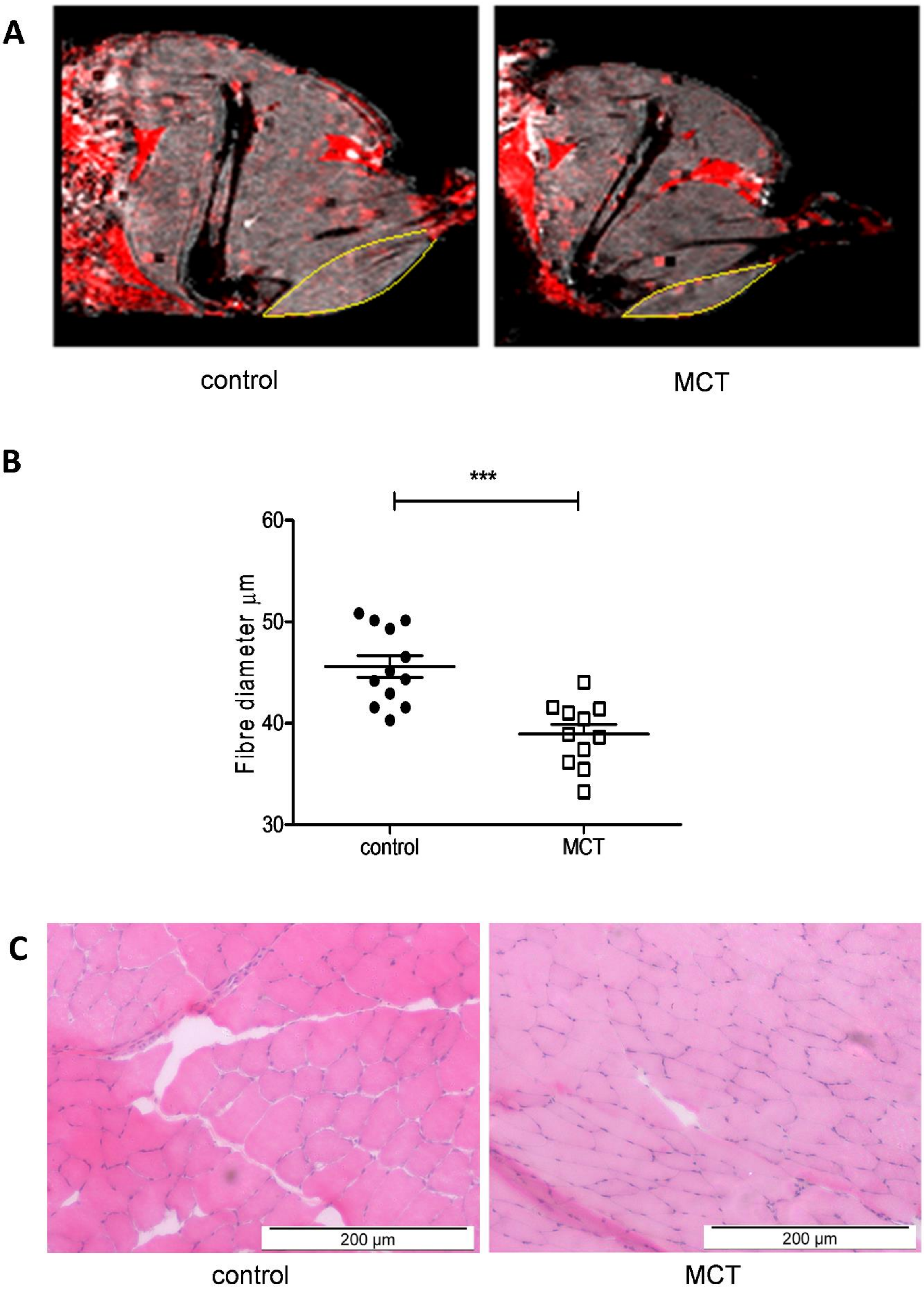
A
control
MCT
B
***
60
50
40
30
Fibre diameter µm
control
MCT
C
200 µm
200 µm
control
MCT

Figure E4. The monocrotaline (MCT) rat is a model of pulmonary hypertension related muscle loss

A. Representative cross sectional MRI image showing the muscle bulk of the hind limb of control and MCT rats taken through the femur with the tibialis anterior outlined in yellow (n=3). **B.** Tibialis anterior (TA) fibre diameter in control (● n=14) and MCT (□ n=16) treated rats (student's t-test $p < 0.001$). **C.** Representative bright-field images of MCT and control rat TA muscle tissue stained with haematoxylin and eosin from which median fibre diameter was determined.

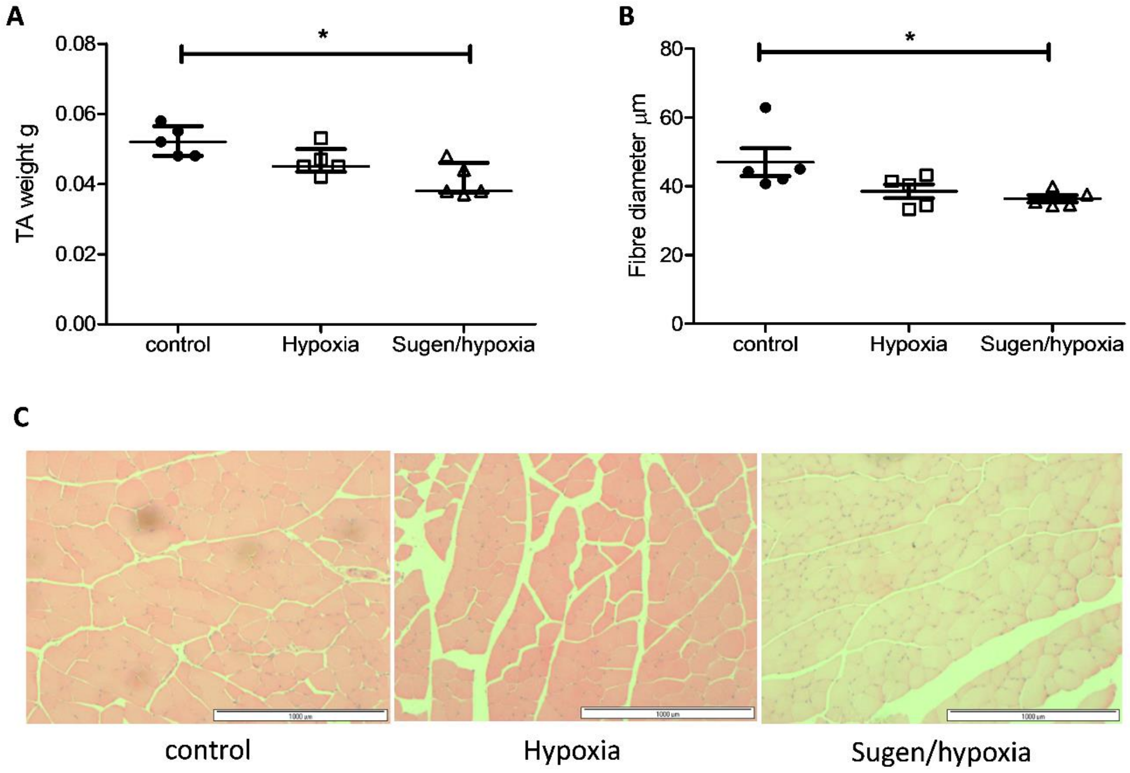
A
0.08
0.06
0.04
0.02
0.00
TA weight g
*
control
Hypoxia
Sugen/hypoxia
B
80
60
40
20
0
Fibre diameter μm
*
control
Hypoxia
Sugen/hypoxia
C
control
Hypoxia
Sugen/hypoxia

Figure E5. The Sugen/hypoxia mouse also exhibits muscle loss

A. Tibialis anterior (TA) muscle weight in control (● n=5), hypoxic (□ n=5) and Sugen/hypoxia (Δ n=5) treated mice (Kruskall-Wallace with Dunn's correction p=0.017). **B.** Tibialis anterior (TA) muscle fibre diameter in control (● n=5), hypoxic (□ n=5) and Sugen/hypoxia (Δ n=5) treated mice (Kruskall-Wallace with Dunn's correction p=0.023). **C.** Representative bright-field images of control, hypoxic and Sugen/hypoxia treated mice TA muscle tissue stained with haematoxylin and eosin from which median fibre diameter was determined.

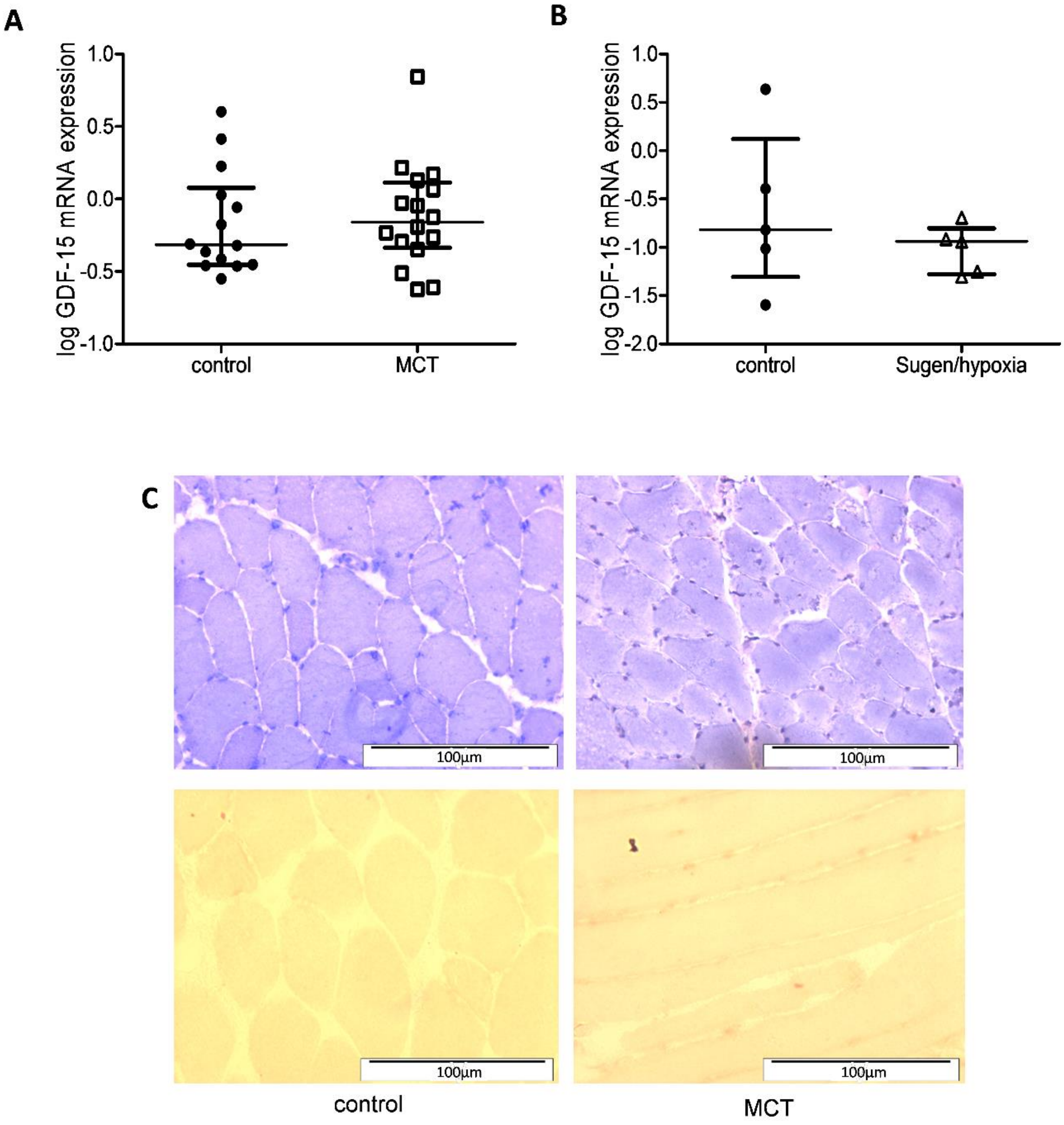
A
log GDF-15 mRNA expression
1.0
0.5
0.0
-0.5
-1.0
control
MCT
B
log GDF-15 mRNA expression
1.0
0.5
0.0
-0.5
-1.0
-1.5
-2.0
control
Sugen/hypoxia
C
100µm
100µm
100µm
100µm
control
MCT

Figure E6. Expression of GDF-15 in the TA of the MCT rat and Sugen/hypoxia mouse

A. log GDF-15 mRNA expression in the TA of control (● n=14) and MCT (□ n=16) treated rats (Mann Whitney U test p =0.575). **B.** log GDF-15 mRNA expression in the TA of control (● n=5) and Sugen/hypoxia (Δ n=5) treated mice (Mann Whitney U test p=0.548) C. Representative Immunohistochemistry of tibialis anterior sections stained for GDF-15 with or without haematoxylin counterstain in control (n=5) and MCT treated rats (n=5).

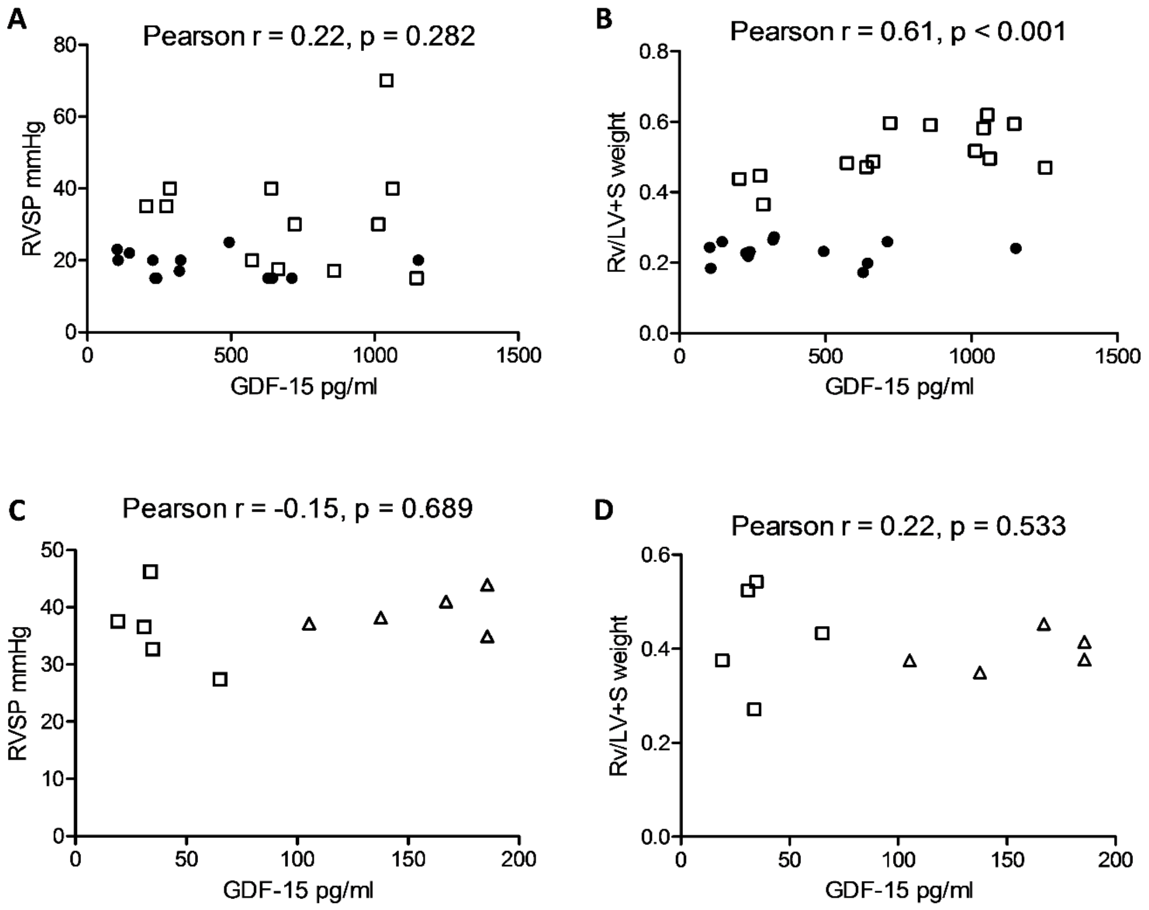
A
Pearson r = 0.22, p = 0.282
RVSP mmHg
GDF-15 pg/ml
B
Pearson r = 0.61, p < 0.001
Rv/LV+S weight
GDF-15 pg/ml
C
Pearson r = -0.15, p = 0.689
RVSP mmHg
GDF-15 pg/ml
D
Pearson r = 0.22, p = 0.533
Rv/LV+S weight
GDF-15 pg/ml

Figure E7. Correlation of circulating GDF-15 with markers of pulmonary pressure and RV hypertrophy in the MCT rat and Sugen/hypoxia mouse.

A. Serum GDF-15 levels plotted against right ventricular systolic pressure (RVSP) (mmHg) in control (● n=14) and MCT (□ n=12) treated rats (Pearson r=0.22 (-0.29 - 0.80), p=0.282). **B.** Serum GDF-15 levels plotted against right ventricle/left ventricle + septal (RV/LV+S) weight in control (● n=14) and MCT (□ n=16) treated rats (Pearson r=0.61 (0.19 - 0.57), p<0.001). **C.** Serum GDF-15 levels plotted against right ventricular systolic pressure (RVSP) (mmHg) in hypoxia (□ n=5) and Sugen/hypoxia (Δ n=5) mice (Pearson r=-0.15 (-0.71 - 0.53), p=0.689). **D.** Serum GDF-15 levels plotted against RV/LV+S weight in hypoxia (□ n=5) and Sugen/hypoxia (Δ n=5) mice (Pearson r=0.22 (-0.47 - 0.75), p=0.533).

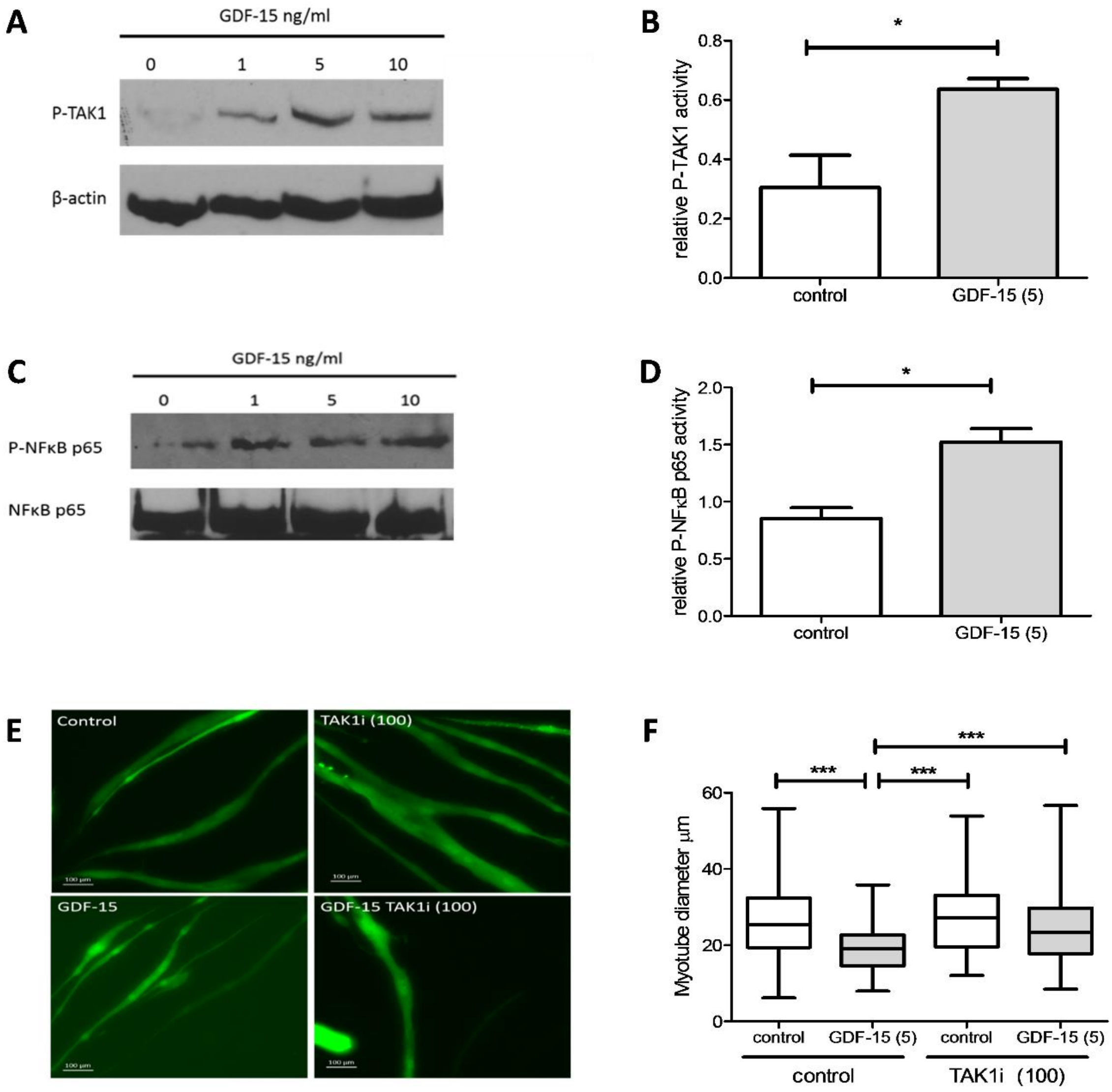
A
GDF-15 ng/ml
0
1
5
10
P-TAK1
β-actin
B
*
relative P-TAK1 activity
0.8
0.6
0.4
0.2
0.0
control
GDF-15 (5)
C
GDF-15 ng/ml
0
1
5
10
P-NFκB p65
NFκB p65
D
*
relative P-NFκB p65 activity
2.0
1.5
1.0
0.5
0.0
control
GDF-15 (5)
E
Control
TAK1i (100)
GDF-15
GDF-15 TAK1i (100)
F
***
***
***
Myotube diameter µm
60
40
20
0
control
GDF-15 (5)
control
GDF-15 (5)
control
TAK1i (100)

Figure E8. GDF-15 at 5ng/ml has the same effects on TAK1, NFκB p65 phosphorylation and myotube diameter as 50ng/ml

Ten day C2C12 myotubes were treated with GDF-15 (0 – 10 ng/ml) for 60 minutes **A.** Phosphorylated TAK1 (P-TAK1) and β actin levels actin in cells treated with 5ng/ml GDF-15 (Representative blot n=3). **B.** Relative intensity of P-TAK1 normalised to β- (Student's t-test p = 0.048). **C.** Phosphorylated p65 NFκB (P-NFκB) and total NFκB levels in cells treated with 5ng/ml GDF-15 (Representative blot n=3). **D.** Relative intensity of P-NFκB normalised to total NFκB (Student's t-test p =0.011). **E.** Representative green fluorescent protein (GFP) fluorescent images of C2C12 myotubes transfected with pCAGGS-EGFP treated with GDF-15 (5ng/ml) with or without the TAK1 inhibitor 5(Z)-7-oxozeanol (100nM) for 48 hours (n=3 experiments). **F.** Diameter of C2C12 myotubes transfected with pCAGGS-EGFP treated with GDF-15 (5ng/ml) with or without the TAK1 inhibitor 5(Z)-7-oxozeanol (100nM) for 48 hours (Kruskall Wallace with Dunn's analysis p < 0.001, n=3 experiments, median number of cells measured per group per experiment 111 (104-117)).

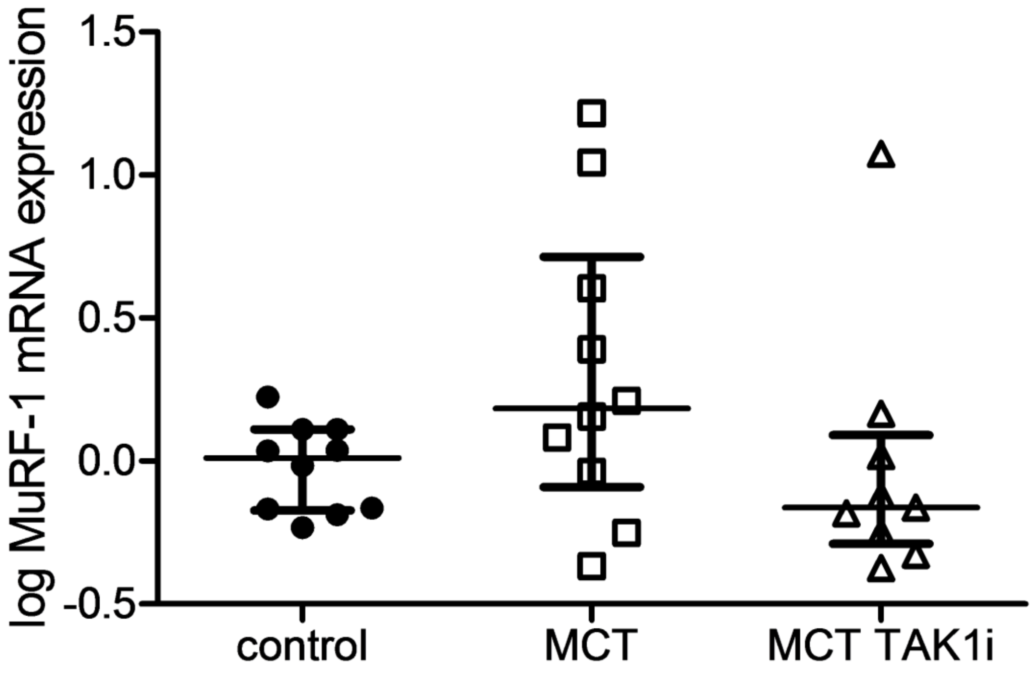
log MuRF-1 mRNA expression
1.5
1.0
0.5
0.0
-0.5
control
MCT
MCT TAK1i

Figure E9. MuRF-1 mRNA expression in the MCT 5(Z)-7-oxozeaenol treated rat.

log MuRF-1 mRNA expression in the TA of control (● n=10), MCT (□ n=10), and MCT TAK1i (Δ n=9) treated rats Kruskall-Wallace with Dunn’s correction (p=0.177).

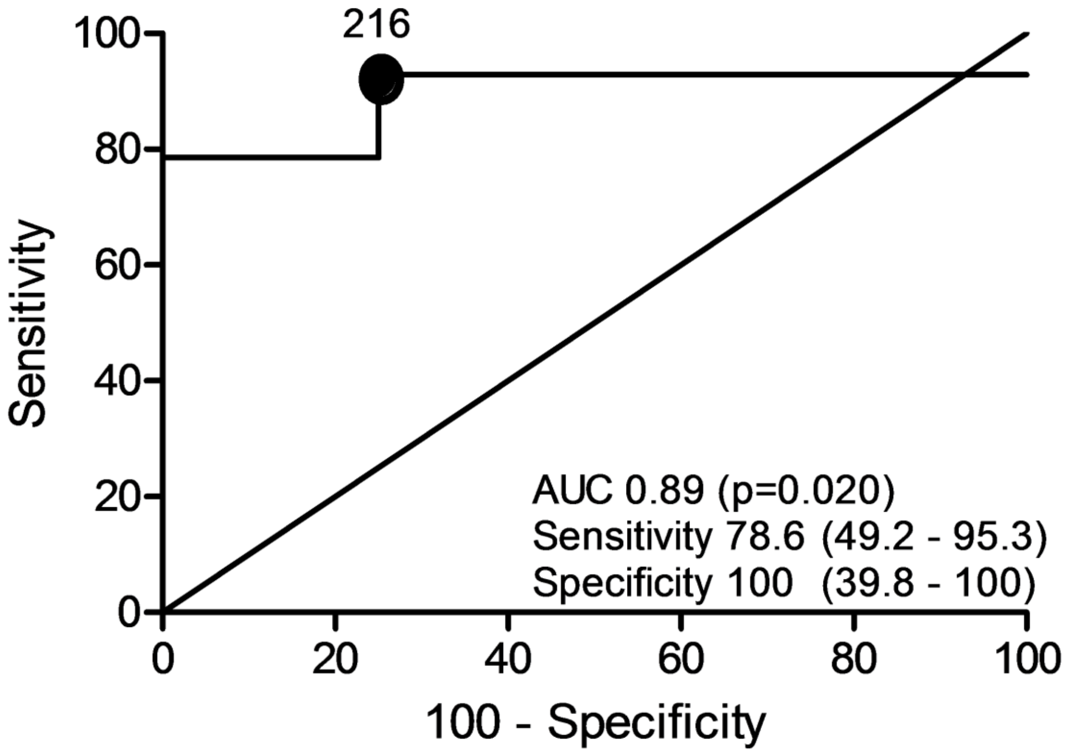

216
100
80
60
40
20
0
Sensitivity
0
20
40
60
80
100
100 - Specificity
AUC 0.89 (p=0.020)
Sensitivity 78.6 (49.2 - 95.3)
Specificity 100 (39.8 - 100)

Figure E10. GDF-15 in the MCT 5(Z)-7-oxozeaenol treated rat.

ROC curve of GDF-15's ability to predict those still growing at the end of the experiment across all animals (n = 18) (AUC 0.89 (0.73 – 1.00) p = 0.020).
